# Timing of singleton births by onset of labour and mode of birth in NHS maternity units in England, 2005–2014: A study of linked birth registration, birth notification, and hospital episode data

**DOI:** 10.1371/journal.pone.0198183

**Published:** 2018-06-14

**Authors:** Peter Martin, Mario Cortina-Borja, Mary Newburn, Gill Harper, Rod Gibson, Miranda Dodwell, Nirupa Dattani, Alison Macfarlane

**Affiliations:** 1 Centre for Maternal and Child Health Research, School of Health Sciences, City, University of London, London, United Kingdom; 2 Population, Policy and Practice Programme, Great Ormond Street Institute of Child Health, University College London, London, United Kingdom; 3 Rod Gibson Associates Ltd., Wotton-under-Edge, United Kingdom; National Academy of Medical Sciences, NEPAL

## Abstract

**Background:**

Maternity care has to be available 24 hours a day, seven days a week. It is known that obstetric intervention can influence the time of birth, but no previous analysis at a national level in England has yet investigated in detail the ways in which the day and time of birth varies by onset of labour and mode of giving birth.

**Method:**

We linked data from birth registration, birth notification, and Maternity Hospital Episode Statistics and analysed 5,093,615 singleton births in NHS maternity units in England from 2005 to 2014. We used descriptive statistics and negative binomial regression models with harmonic terms to establish how patterns of timing of birth vary by onset of labour, mode of giving birth and gestational age.

**Results:**

The timing of birth by time of day and day of the week varies considerably by onset of labour and mode of birth. Spontaneous births after spontaneous onset are more likely to occur between midnight and 6am than at other times of day, and are also slightly more likely on weekdays than at weekends and on public holidays. Elective caesarean births are concentrated onto weekday mornings. Births after induced labours are more likely to occur at hours around midnight on Tuesdays to Saturdays and on days before a public holiday period, than on Sundays, Mondays and during or just after a public holiday.

**Conclusion:**

The timing of births varies by onset of labour and mode of birth and these patterns have implications for midwifery and medical staffing. Further research is needed to understand the processes behind these findings.

## Introduction

The times at which services are available have taken a prominent place in policies about the National Health Service (NHS) in England. The aims set out in the Five Year Forward plan for the NHS in England included ‘Ensuring that hospital patients have access to seven day services where this makes a clinical difference to outcomes’ [1]. Care in labour and at birth is one of the services that has always been provided 24 hours a day, on all seven days of the week. Several recent studies have analysed the outcome of pregnancy by time of day and day of the week of birth in Scotland [2], England [3,4] and the Netherlands [5], but none has included an in-depth analysis of how the time of birth varies with the onset of labour and mode of birth.

A study of daily variations in numbers of births in England and Wales in the 1970s found a pronounced weekly cycle, with the daily maximum numbers of births occurring between Tuesday and Friday, while fewer births occurred on Mondays and Saturdays, and fewest on Sundays. Numbers of births were also low on public holidays, with Christmas Day and Boxing Day having the lowest numbers of births in each year [6,7]. Broadly similar patterns were observed in more recent data [8,9] and in other countries [10–17]. Studies from England and Wales in the late 1970s [18], England in the 1990s and early 2000s [19], and also in France [15], Australia [14] and Israel [20] showed that elective caesarean births and, to a lesser extent, births following induction were more likely to occur on weekdays than at weekends and made a major contribution to the daily variations in numbers of births.

Most of these analyses did not include data about the time of day of birth. The time of birth was not recorded in national routine data systems in England and Wales until 2005, for example. On the other hand, it has long been established that numbers of spontaneous births vary by time of day and are higher at night than during the day [21–25], with numbers of births after spontaneous labour being highest between 1 am and 7 am. It has been suggested that this pattern is a residual of human evolutionary heritage. For animals that live in groups that are mostly active and often dispersed during the day, and come together to rest at night, a night-time onset of labour and birth in the early hours of the morning mean that the mother and newborn baby can expect to receive some protection from predators [23,26].

Early evidence was provided by Adolphe Quetelet in his ‘Essai de physique sociale’ [22] published in 1835. This cited data from Brussels and Hamburg to show that the numbers of live births in the six hour periods before and after midnight were higher than those in the corresponding periods before and after noon. A recent analysis of births in a Madrid maternity hospital between 1887 and 1892 found that 98% of these births occurred without intervention, and that the number of births was higher between 4am and noon than at other periods of the day [26]. Analyses of births in New York State in 1929 and 1936 [24] found that numbers of spontaneous births were highest in the early hours of the morning from 3-5am while numbers births involving obstetric intervention peaked from 9-11am. A study of over 16,000 births in Birmingham, England in the early 1950s also analysed the time of onset of spontaneous labour and found that this peaked in the middle of the night, around 2am, with numbers of births peaking from 3-5am. [25]. Some of the rationale for the rising rates of induction, augmentation and caesarean birth in the 1970s was to concentrate births into conventional working hours in order to optimise access to medical care [27–29].

High rates of obstetric intervention have been shown to alter the circadian rhythms of births. In a recent study in Spain, births without intervention were found to follow a roughly sinusoidal curve, with the familiar night-time peak, but a high rate of caesarean births in day time meant that overall the majority of births occurred during the day [30]. In the United States, analysis of births in 41 states plus the District of Columbia in 2013 showed patterns which were very different from those observed in the mid twentieth century [31]. Hospital births were concentrated into daytime and early evening hours, although there were variations by mode of birth [31].

A study of data from the 1990 National Perinatal Database in the Netherlands found that patterns of birth times of vertex singleton term births without oxytocic drugs depended on who provided care–midwives or obstetricians [32]. In women cared for by midwives, numbers of births to both primiparous and multiparous women peaked earlier in the day than those to women cared for by obstetricians.

The aim of this paper is to describe in detail how day and time of birth vary by onset of labour, mode of birth, and gestational age in NHS maternity units in England. This is the first study to use a national data set to analyse numbers of births by hour of birth in England.

## Methods

### Data

This study used linked data from birth registration, birth notification, and Maternity Hospital Episode Statistics (HES) for singleton births in England from 1^st^ January 2005 to 31^st^ December 2014. The data were derived from a larger dataset linking all births in England and Wales during this period. After piloting in an earlier collaborative project [33], linkage of birth registration and birth notification data had been mainstreamed by the Office for National Statistics (ONS), which provided linked birth registration and birth notification records relating to 7,013,804 births in England and Wales from 2005 to 2014 for this study. Data about the 6,676,912 births in England were linked to Maternity HES delivery records by the Health and Social Care Information Centre (HSCIC), now known as NHS Digital, using a slightly modified version of an in-house algorithm [34,35]. Singleton and multiple births were then separated for quality assurance and analysis [36]. The work was undertaken in the secure environment of the Office for National Statistics’ Secure Research Service, previously known as the Virtual Microdata Laboratory (VML).

**Fig 1:**
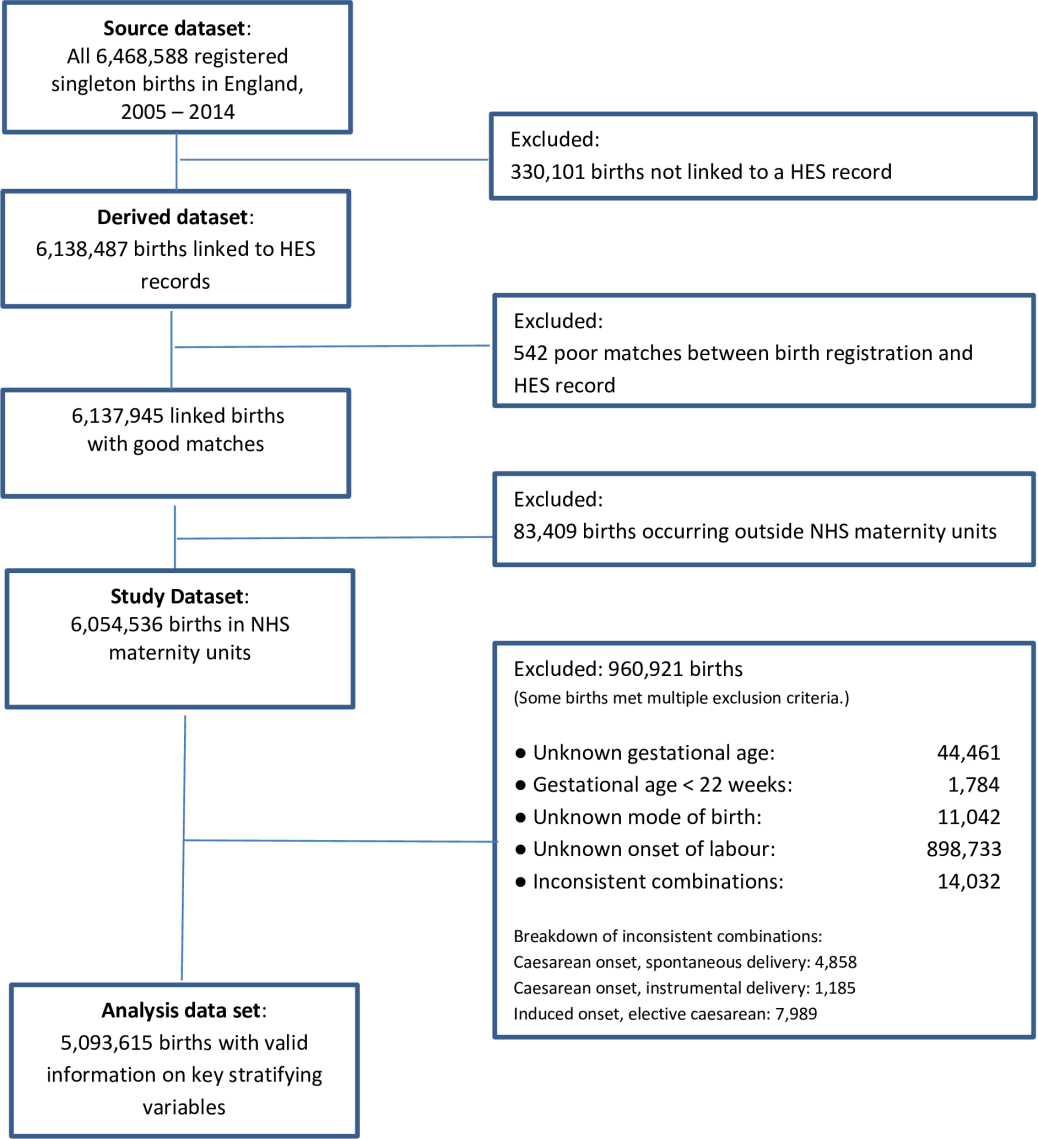
Flowchart: How we derived the dataset for analysis.

The steps we took to derive a data set for analysis are illustrated in Fig 1. Our source dataset consisted of all 6,468,588 singleton births occurring from 1 January 2005 to 31 December 2014 registered in England. After linkage and quality assurance, 6,138,487 singleton births were judged to be linked to the correct HES delivery record. We then excluded 542 additional records because, after a careful comparison of the records, we were not confident that the linkage between birth registration and birth notification was correct. Our resulting dataset contained 6,137,945 records, 94.9% of the source dataset. We investigated whether the births in this dataset differed from the source dataset with respect to region of residence, year, month, type of day and hour of birth, age of mother, sex and gestational age of baby. For each of these variables, there was a statistically significant difference between the distributions of births included in and excluded from the derived dataset population. Births from later years were more likely to be linked than those from earlier years. The differences between the source dataset and the derived dataset were small, however, below one percentage point for any category. The analyses are reported in detail in S2 Appendix.

To derive our study dataset, we included only the 6,054,536 births recorded as having taken place in NHS maternity units, excluding 83,409 births that took place at home, in private hospitals, in NHS hospitals without maternity facilities, or elsewhere outside a hospital. Births outside NHS hospitals, including those occurring at home have low rates of inclusion in Maternity HES and as a consequence, low rates of linkage, so overall data quality would have suffered had we included them in the analysis.

Finally, we excluded 960,921 births records most of which had missing or inconsistent data about the onset of labour, a key variable, and a few of which had mode of birth or gestational age missing. The excluded births made up 15.9% of the records, leaving data for 5,093,615 births. We investigated whether the exclusion of records with missing information changed the distribution of births with respect to region of residence, year, month, type of day and hour of birth, age of mother, sex and gestational age of baby. We found statistically significant differences between included and excluded births for all variables, except the baby’s sex. Nonetheless, differences between all linked NHS hospital births and our analysis data set were small, the biggest difference being 1.02 percentage points for a category. These analyses are reported in detail in S3 Appendix. Data completeness improved over the study period: in the years from 2005–2008, we had to exclude between 12.0% and 16.6% of records per year, whereas in the years from 2009 to 2014, only between 6.8% and 9.0% of records had to be excluded per year. The improving data completeness of Maternity Hospital Episode records over this period has been noted by others [37,38].

### Variables

#### Onset of labour

The onset of labour is recorded in the HES delivery record maternity tail in the variable ‘DELONSET’. We grouped codes for onset of labour into three categories, spontaneous, induced, or no labour (caesarean), as shown in S1 Appendix. For the small proportion of births with several HES records per delivery, the record containing information on onset of labour was used.

#### Mode of birth

Mode of birth was derived from the procedure codes in the core admitted patient care record using the Office of Population Censuses and Surveys (OPCS-4) Classification of Interventions and Procedures, versions 4.2 to 4.7. These procedure codes are submitted to the HES database by local NHS providers, who in turn derive the codes from locally held clinical notes. We coded mode of birth as spontaneous, instrumental, elective caesarean, or emergency caesarean. Details of the derivation are described in S1 Appendix.

#### Gestational age

Birth notification records gestation length in completed weeks since the first day of the last menstrual period. We coded gestational age into three categories: pre-term (22–36 weeks), term (37–41 weeks), and post-term (42 weeks or more).

#### Time of birth

At birth notification, the time of birth is recorded as hour and minute of the day. We grouped this into hourly intervals, with hours running from 0 to 23, where 0 means 0:00 hrs to 0:59 hrs. The time of birth was missing for 45,364 births in the analysis data set. Of these missing values, 43,414 related to births in October 2008; times were available for only eight births during this month. Births whose times are missing were excluded from analyses involving the time of birth, but were included in analyses involving the day of birth only.

#### Day of birth

Day of birth was derived using the date of birth recorded at birth registration, which is the most complete and accurate record of dates of birth in England and Wales. Dates of birth were missing from 16.9% of Maternity HES records. Although the dates of all births are recorded at birth notification, it is not known whether any quality checks are carried out. Pilot linkage of birth notification data to birth registration data for 2005 had shown that the date of birth was discordant in only 0.3 per cent of the linked records [33].

#### Type of day

For descriptive analyses, we grouped the day of the week into eight categories: non-holiday days of the week from Monday to Sunday, and public holidays which included bank holidays given in lieu of public holidays that occurred at weekends. For the purpose of statistical modelling, we constructed a variable ‘type of day’ that distinguishes eleven types of day, including the seven days of the week, and in addition:

- Holiday: bank holidays, including those given in lieu of public holidays falling on a weekend, but excluding 25 and 26 December)
- Christmas: 25 and 26 December
- Day before holiday: the last weekday before a holiday period e.g. Maundy Thursday
- Day after holiday: the first weekday after a holiday period, e.g. the Tuesday after Easter.

Categories were mutually exclusive; for example, Good Friday was categorised as a holiday rather than as a Friday.

### Statistical analysis

#### Descriptive analysis

We performed descriptive analyses of time of birth and day of the week separately for eight combinations of onset of labour and mode of birth, as shown in Table 1. In descriptive figures, each data point represents the mean number of births in a particular hour of the week. For example, “Monday 0:00 to 0:59” records the mean number of births in the first hour of a non-holiday Monday. The mean number of births is a suitable statistic, because it allows direct comparison between the mean number of births per hour on different types of day, taking into account differences in numbers of each type of day in the study period. For example, there were more non-holiday Tuesdays than non-holiday Mondays.

**Table 1:** Numbers of singleton births in NHS maternity units by onset of labour, mode of birth, and gestational age group, 2005–2014.

Onset of labour	Mode of birth	Gestational Age			Total	Percentage
		Term	Pre-term	Post-term		
**Spontaneous**	**Spontaneous**	2,401,010	121,915	45,487	**2,568,412**	***50*.*4***
	**Emergency caesarean**	308,248	29,440	11,121	**348,809**	***6*.*8***
	**Instrumental**	425,955	21,542	12,472	**459,969**	***9*.*0***
**Induced**	**Spontaneous**	577,544	38,249	60,075	**675,868**	***13*.*3***
	**Emergency caesarean**	188,413	11,913	38,335	**238,661**	***4*.*7***
	**Instrumental**	162,882	7,168	28,006	**198,056**	***3*.*9***
No labour*	Elective caesarean*	441,500	23,879	4,614	**469,993**	***9*.*2***
	**Emergency caesarean**	84,384	44,465	4,998	**133,847**	***2*.*6***
**Total**		**4,589,936**	**298,571**	**205,108**	**5,093,615**	***100*.*0***
**Percentage**		***90*.*1***	***5*.*9***	***4*.*0***	***100*.*0***	

*Among the elective caesarean births, 445,543 were recorded as caesarean onset, the remaining 24,450 were recorded as ‘spontaneous onset’. We combined these two categories, since the latter group was too small for meaningful estimation.

#### Statistical modelling

There were 3652 days from 1 Jan 2005 to 31 Dec 2014. Our model aimed to predict the number of births on each of these days, separately for each of eight combinations of onset of labour and mode of birth. To account for overdispersion, we fitted negative binomial regression models with a logarithmic link, and included the following predictor variables:

- Type of day as described above
- Harmonic terms to account for the yearly cycle of birth frequencies [39]. This is important, as without this adjustment our model would not be able to distinguish seasonal variations in birth frequencies from the effects of holidays, such as Christmas. We used a periodogram [40] to identify relevant cyclical frequencies, and set up the harmonic terms as follows: the days of the year from 1 January to 31 December were transformed into angles on the radian scale, from 0 to just under 2π. The sine and cosine of the angles were then added as predictor variables in the model, at all frequencies from 1 to 9 (where 1 signifies a 12-months cycle, and 9 signifies a 1.33-months-cycle).
- A natural cubic spline term to control for trends over time, estimated using the 120 months from Jan 2005 to Dec 2014, where the months were coded sequentially 0 to 119. We allowed 3 degrees of freedom per year. Without this adjustment, trends in numbers of births and trends in intervention rates over our study period could have confounded our estimates of holiday effects.
- Two dummy variables identifying days of clock changes. Days on which the clock changes to British Summer Time have 23 hours and thus a lower expected number of births; days on which the clock changes back to Greenwich Mean Time have 25 hours and a higher expected number of births.

Estimates are presented as rate ratios (RR): the ratio of the expected number of births on one type of day over the mean number of births per day. For example, a RR of 1.10 for a Friday means that the expected number of births on Fridays is 10% higher than the mean, and a RR of 0.88 for a Sunday means that the expected number of births on Sundays is 12% lower than the mean. All rate ratios are adjusted for yearly cycles, trend, and clock changes, as described above. Model comparisons were performed using likelihood ratio tests.

We calculated 99% confidence intervals around parameter estimates, and for hypothesis tests used a significance level of α = 0.01. All statistical analyses were carried out in R, version 3.1.2 [41].

#### Approvals

Ethics approval 05/Q0603/108 and subsequent substantial amendments were granted by East London and City Local Research Ethics Committee 1 and its successors.

Permission to use confidential patient information without consent under Section 60 of the Health and Social Care Act 2001 was initially granted by the Patient Information Advisory Group PIAG 2-10(g)/2005. Renewals and amendments under Regulation 5 of the Health Service (Control of Patient Information) Regulations 2002 were granted by its successor bodies, the National Information Governance Board and the Health Research Authority.

A second permission CAG 9-08(b)2014 to use confidential patient information without consent under Regulation 5 of the Health Service (Control of Patient Information) Regulations 2002 to create a research database held at the Office for National Statistics for analyses relating to inequalities in the outcome of pregnancy and to inform maternity service users about the outcome of midwifery, obstetric and neonatal care was granted by the Health Research Authority.

Permission to access data from the Office for National Statistics in the VML, now known as the Secure Research Service, was granted by ONS’s Microdata Release Panel. All members of the research team successfully applied for ONS Approved Researcher Status.

Permission to link and analyse data held by the Health and Social Care Information Centre, now NHS Digital, was granted under Data Sharing Agreement NIC-273840-N0N0N.

## Results

### Daily and weekly patterns of births by onset of labour and mode of birth

Table 1 shows the number of births by onset of labour, mode of birth, and gestational age group.

**Fig 2:**
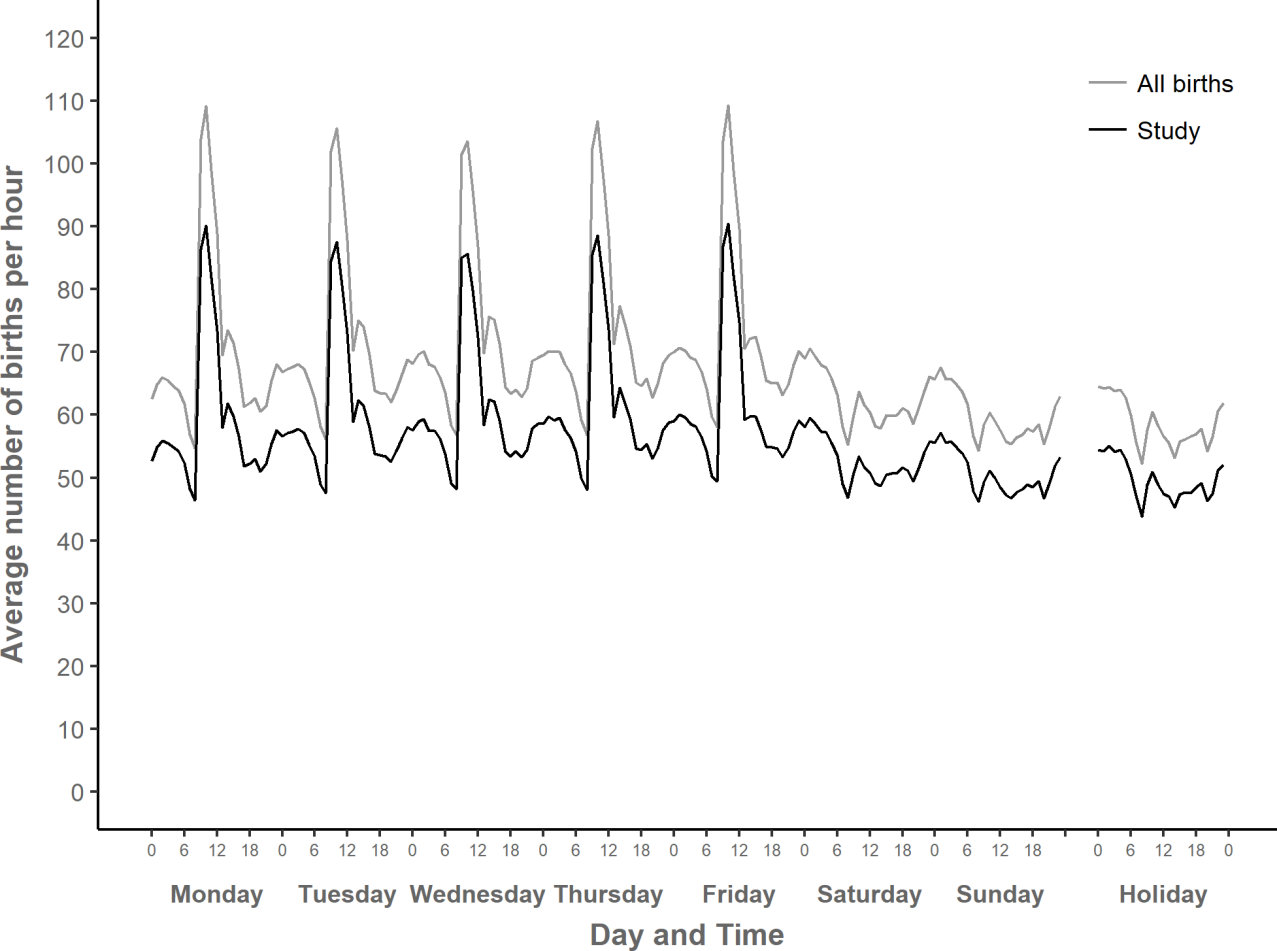
Mean number of singleton births in NHS maternity units per hour over the week, England 2005–2014. “All births”: All singleton births in NHS maternity units in England linked in this study, 2005–2014: *N* = 5,999,732 (missing times: 54,804); “Study”: Singleton births included in study: *n* = 5,048,269 (missing times: 45,346).

Fig 2 shows the average number of births per hour in NHS maternity units in England over the course of the week and on public holidays. The pattern is the same, whether we look at all singleton births in NHS maternity units in England, shown with a grey line, or the somewhat smaller subset of births for which we have complete data and which could be included in our analyses, shown with a black line (see Fig 1 for an explanation of how these two sets of births differ). This suggests that our study dataset is representative of the timing of births in NHS maternity units in England from 2005 to 2014.

In Fig 2, the peaks in mean numbers of births occur on weekdays between 9:00 and 12:59, with a smaller secondary peak between 14:00 and 14:59 on weekdays. A second period of relatively high numbers of births, albeit with a much smaller peak, occurs between 22:00 and 6:59 at night, regardless of the type of day. Low points are between 8:00 and 8:59, on weekdays and also at weekends and on holidays.

It is relevant to consider the proportion of births that occur outside of what are considered to be usual working hours. In our study data set, 71.5 per cent of births occurred out of hours, that is at weekends, on holidays, or on weekdays between 17:00 and 8:59. Conversely, 28.5 per cent of births occurred within usual working hours, that is on weekdays between 9:00 and 16:59. Among all singleton births in NHS maternity units, 71.4 per cent occurred out of hours, and 28.6 per cent within usual working hours.

**Fig 3:**
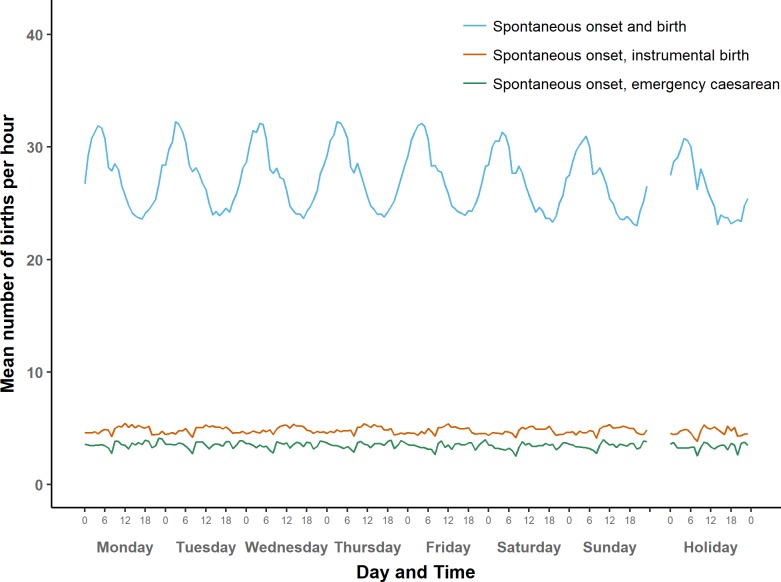
Average number of term births per hour by type of day: Spontaneous onset of labour.

**Fig 4:**
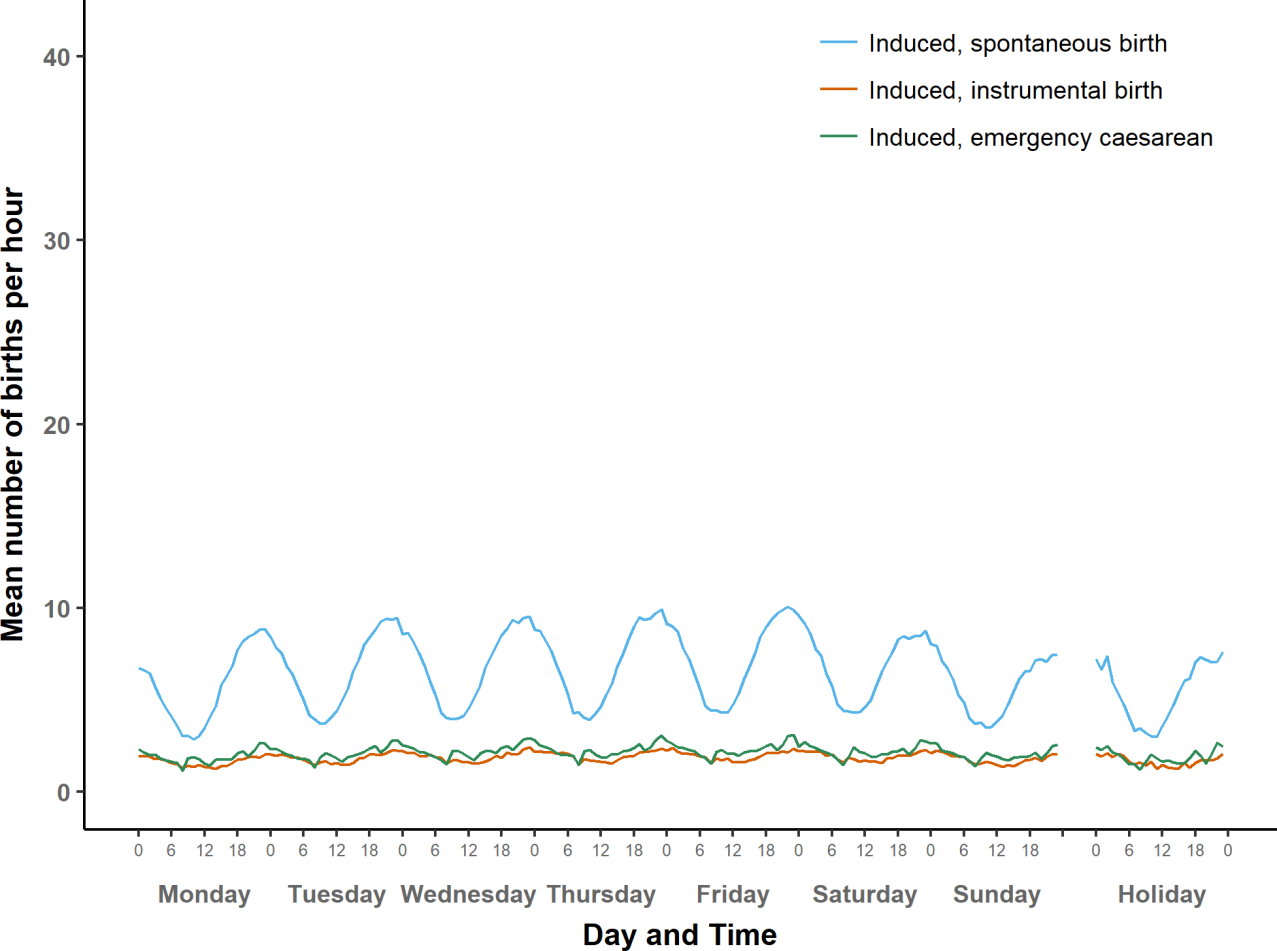
Average number of term births per hour by type of day: Induced births.

**Fig 5:**
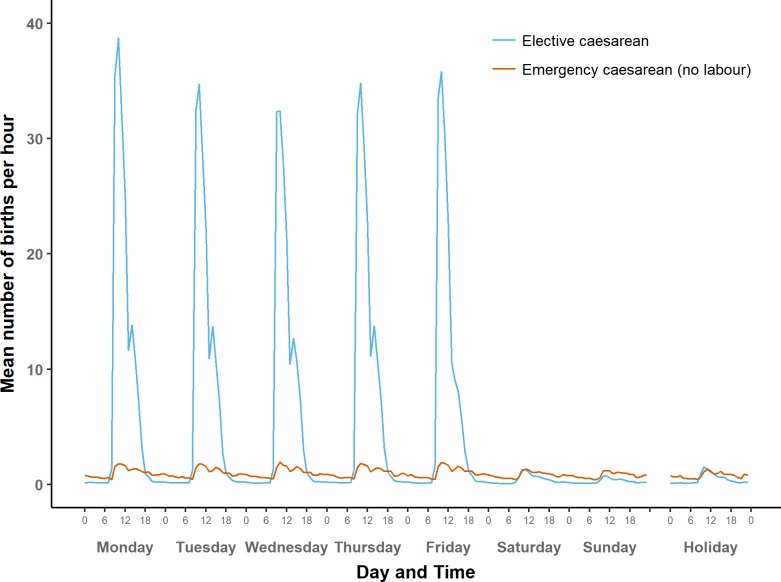
Average number of term births per hour by type of day: Caesarean births without prior onset of labour.

**Fig 6:**
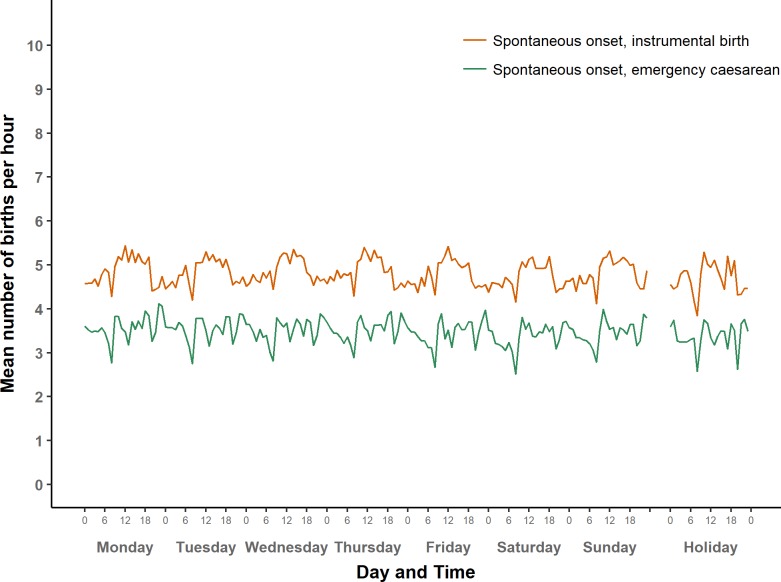
Average number of term births per hour by type of day: Spontaneous onset of labour (larger scale).

**Fig 7:**
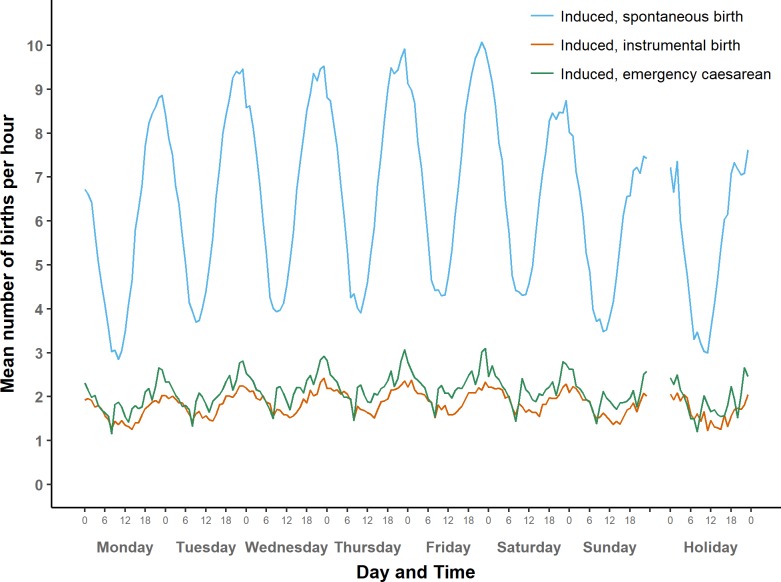
Average number of term births per hour by type of day: Induced births (larger scale).

While Fig 2 shows the overall daily and weekly cycles of births, Figs 3–7 demonstrate how these cycles differ by onset of labour and mode of birth. There are clear differences in circadian and weekly patterns. S4 Appendix shows a more detailed view of average numbers of births per hour in each of the eight combinations of onset labour and mode of birth, for a selected weekday (Thursday). Graphical data exploration (not shown here) suggested that the circadian patterns did not vary appreciably by gestational age group so this article focussed on circadian patterns in term births, but weekly patterns of births were analysed by gestational age group.

#### Spontaneous onset

Fig 3 shows the average numbers of births with spontaneous onset over the course of a week, by mode of birth. Fig 6 then presents instrumental births and emergency caesareans on a larger scale to show their patterns more clearly. Just over half of all births in our data set are spontaneous births following spontaneous onset of labour. These have a roughly sinusoidal pattern, and are most likely to occur between 1:00 and 7:00, with a peak around 4:00, and a trough in the afternoon. In addition, there is a small but noticeable interruption of the sinusoidal pattern at 8:00.

In contrast, average numbers of instrumental births after spontaneous onset of labour are higher between 9:00 and 17:00 than in the night and early morning. The lowest hourly numbers of births in this group was from 8:00–8.59. Numbers of births where spontaneous onset was followed by an emergency caesarean were higher between 21:00 and midnight than in the early hours of the morning. There are three distinct dips in the numbers, most pronounced at 8:00, and somewhat less deep at 13:00 hrs and 20:00.

In general, the variations in numbers of births after spontaneous onset over the course of day did not vary much between weekdays, weekends, and holidays. Nonetheless, numbers of spontaneous births after spontaneous onset were slightly lower on Sundays and holidays compared to weekdays, as we will explore in detail below.

#### Induced onset

Fig 4 shows the average numbers of induced births over the course of a week, by mode of birth. Fig 7 shows the same on a larger scale. Numbers of spontaneous births after induced onset follow a sinusoidal pattern, with a peak during the hour before midnight and a trough just before noon.

The same midnight peak appears when inductions are followed by instrumental births or emergency caesareans, although in these groups the patterns are less regular than for induced spontaneous births. The three groups of induced births share a weekly cycle that has its highest peak on Friday night and its trough on Monday morning.

#### Caesarean births

Fig 5 shows the distribution of caesarean births without prior onset of labour, separately for elective and emergency caesareans. Elective caesareans are mostly carried out on weekdays between 9:00 and 12:00, with a pronounced peak between 9:00 and 10:59. Very few occur between 17:00 and 7:00 on weekday evenings and nights, and even fewer at any time at weekends and on holidays.

Emergency caesareans without prior onset of labour are distributed somewhat similarly to elective caesareans, with a pronounced peak between 9:00 and 12:00. However, mean numbers of emergency caesareans without prior onset of labour are higher than mean numbers of elective caesareans in the evening, at night and in the early morning, and the same applies to weekends and holidays.

#### Day of the week differences

We fitted statistical models to estimate differences in the daily numbers of term births by type of day, separately for each of the eight groups, adjusted for yearly cycles in numbers of births and trends over time. Results are presented in Table 2. The models fitted well. Residuals were approximately normally distributed for all models, and there were no influential outliers. Adjusted *R*^*2*^ values ranged from 0.42 (spontaneous onset and emergency caesarean) to 0.97 (elective caesarean). Differences by type of day were statistically significant (*p* < .001) for all groups, but their strength varied considerably. Type of day accounted for 94.6% of the variation in daily frequencies among elective caesarean births, but only for 0.4% of the variation among instrumental births after spontaneous onset of labour.

**Table 2:** Adjusted rate ratios for frequency of term births by day of the week, compared to the overall average, by onset of labour and mode of birth (99% confidence intervals in brackets).

Onset	Spontaneous						Induced						No labour			
Mode	Spontaneous		Emergency CS		Instrumental		Spontaneous		Emergency CS		Instrumental		Elective CS		Emergency CS	
Mon	**1.00**	(0.99, 1.00)	**1.02**	(1.01, 1.04)	**1.01**	(0.99, 1.02)	**0.87**	(0.86, 0.88)	**0.89**	(0.87, 0.90)	**0.88**	(0.87, 0.90)	**1.53**	(1.52, 1.55)	**1.05**	(1.01, 1.09)
Tues	**1.01**	(1.00, 1.01)	**1.02**	(1.00, 1.03)	**1.00**	(0.99, 1.01)	**1.02**	(1.01, 1.03)	**0.98**	(0.97, 1.00)	**0.99**	(0.97, 1.01)	**1.38**	(1.37, 1.40)	**1.06**	(1.02, 1.09)
Wed	**1.01**	(1.01, 1.02)	**1.01**	(1.00, 1.02)	**1.01**	(1.00, 1.02)	**1.05**	(1.04, 1.06)	**1.04**	(1.02, 1.05)	**1.03**	(1.01, 1.05)	**1.36**	(1.34, 1.37)	**1.07**	(1.03, 1.10)
Thurs	**1.01**	(1.01, 1.02)	**1.01**	(1.00, 1.02)	**1.01**	(1.00, 1.02)	**1.07**	(1.06, 1.08)	**1.06**	(1.04, 1.07)	**1.06**	(1.04, 1.08)	**1.41**	(1.40, 1.43)	**1.08**	(1.05, 1.12)
Fri	**1.01**	(1.01, 1.02)	**0.99**	(0.98, 1.01)	**1.00**	(0.99, 1.01)	**1.10**	(1.09, 1.11)	**1.09**	(1.07, 1.10)	**1.06**	(1.04, 1.07)	**1.36**	(1.35, 1.38)	**1.09**	(1.06, 1.13)
Sat	**0.99**	(0.99, 1.00)	**0.97**	(0.95, 0.98)	**0.98**	(0.97, 0.99)	**1.04**	(1.03, 1.05)	**1.03**	(1.01, 1.05)	**1.04**	(1.03, 1.06)	**0.08**	(0.08, 0.09)	**0.86**	(0.84, 0.89)
Sun	**0.98**	(0.97, 0.98)	**0.99**	(0.98, 1.00)	**1.00**	(0.99, 1.01)	**0.88**	(0.87, 0.89)	**0.95**	(0.93, 0.96)	**0.96**	(0.94, 0.97)	**0.06**	(0.05, 0.06)	**0.83**	(0.80, 0.86)
Holiday	**0.98**	(0.97, 0.99)	**0.99**	(0.96, 1.02)	**0.98**	(0.96, 1.01)	**0.88**	(0.86, 0.91)	**0.92**	(0.88, 0.96)	**0.92**	(0.88, 0.97)	**0.09**	(0.08, 0.10)	**0.84**	(0.76, 0.92)
Christmas	**0.93**	(0.90, 0.95)	**0.88**	(0.82, 0.95)	**0.97**	(0.92, 1.03)	**0.61**	(0.57, 0.65)	**0.79**	(0.72, 0.87)	**0.81**	(0.74, 0.90)	**0.05**	(0.04, 0.06)	**0.72**	(0.58, 0.88)
Before holidayolHoldhOLD	**1.00**	(0.98, 1.01)	**0.96**	(0.92, 1.00)	**1.00**	(0.97, 1.04)	**1.10**	(1.06, 1.13)	**1.08**	(1.03, 1.13)	**1.08**	(1.03, 1.14)	**1.43**	(1.39, 1.48)	**1.11**	(1.01, 1.21)
After holiday	**0.99**	(0.97, 1.00)	**1.02**	(0.99, 1.06)	**1.00**	(0.97, 1.03)	**0.87**	(0.84, 0.90)	**0.86**	(0.82, 0.91)	**0.88**	(0.83, 0.93)	**1.62**	(1.58, 1.67)	**0.99**	(0.90, 1.10)
Adj. *R*^2^	0.843		0.423		0.623		0.826		0.664		0.737		0.972		0.628	
% Dev	1.5%		1.9%		0.4%		18.7%		7.2%		4.6%		94.6%		9.3%	
*N*	2,401,010		308,248		425,955		577,544		188,413		162,882		441,500		84,384	

Adjusted rate ratios compare the predicted number of births on each type of day to the overall average, within each group (column), after adjusting for yearly cycle and trend. In all models, ‘type of day’ was statistically significant with *p* < .001 (likelihood ratio test, df = 10). Before Holiday: the last weekday before a holiday period. After Holiday: the first weekday after a holiday period. Adj *R*^2^: Adjusted *R*^2^ value for the whole model. % Dev: the percentage of total deviance explained by type of day. CS: caesarean section.

Table 2 also presents estimated adjusted rate ratios for differences by type of day in the numbers of term births. For births after spontaneous onset of labour, differences by type of day are small. Numbers of spontaneous births after spontaneous onset are higher on weekdays, especially from Wednesday to Friday, than on Sundays and holidays We estimated that seven per cent fewer babies are born. on Christmas Day and Boxing Day, compared to an average day. Similar, but smaller, differences between types of day were found for emergency caesareans and instrumental births after spontaneous onset.

As we have already seen in Fig 3, numbers of births after induction of labour differ more strongly by type of day than births after spontaneous onset. There is a weekly cycle with a peak on Friday and a trough on Sunday and Monday. Holidays and Sundays have similar rates but there are far fewer induced births on Christmas Day and Boxing Day than any other type of day. Like Mondays, days after holidays have low numbers while days before holidays are similar to Fridays in having high rates. This general pattern is the same for all induced births, although the size of these differences varies by mode of birth, with the widest differences between days being among spontaneous births.

Differences between days are most pronounced among elective caesarean births, which rarely occur at weekends or on public holidays. Numbers of elective caesareans tend to be highest on Mondays and on weekdays after a holiday period followed by Thursdays and weekdays before a holiday, and then Tuesday, Friday, and Wednesday.

Emergency caesareans without prior onset of labour are also more frequent on weekdays compared to weekends and holidays, although the differences are small compared to those in elective caesareans. Numbers of emergency caesareans without prior labour are highest on Mondays, Tuesdays and weekdays after holidays.

### Differences between pre-term, term, and post-term births

**Fig 8:**
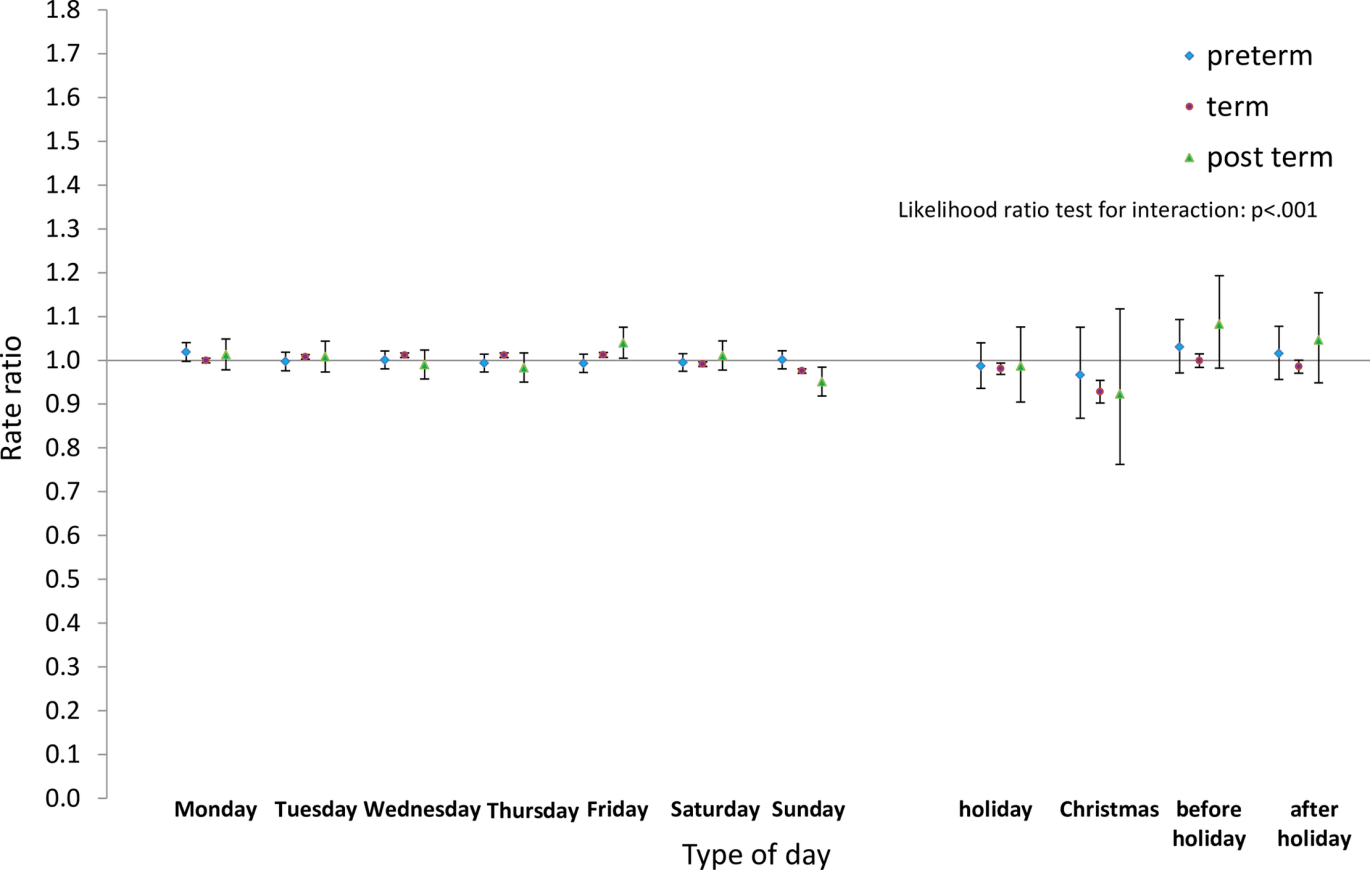
Rate ratios and confidence intervals for type of day differences by gestational age: Spontaneous onset and birth. Vertical axes show rate ratios relative to the overall average for each gestational age group within each figure. Error bars show 99% confidence intervals. Likelihood Ratio Test of H_0_: Type of day differences do not differ between term, pre-term and post-term births.

**Fig 9:**
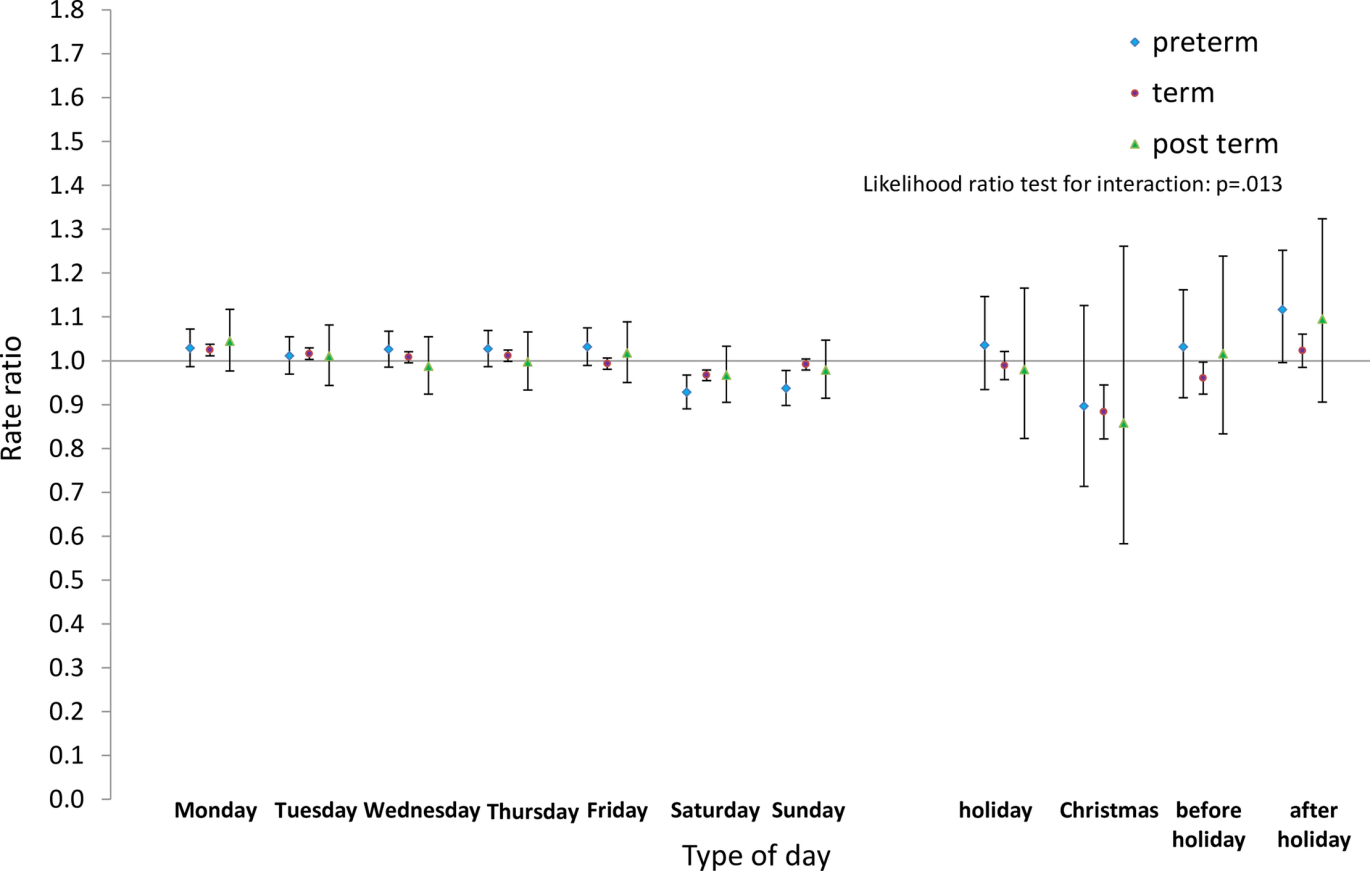
Rate ratios and confidence intervals for type of day differences by gestational age: Spontaneous onset, emergency caesarean. Vertical axes show rate ratios relative to the overall average for each gestational age group within each figure. Error bars show 99% confidence intervals. Likelihood Ratio Test of *H*_0_: Type of day differences do not differ between term, pre-term and post-term births.

**Fig 10:**
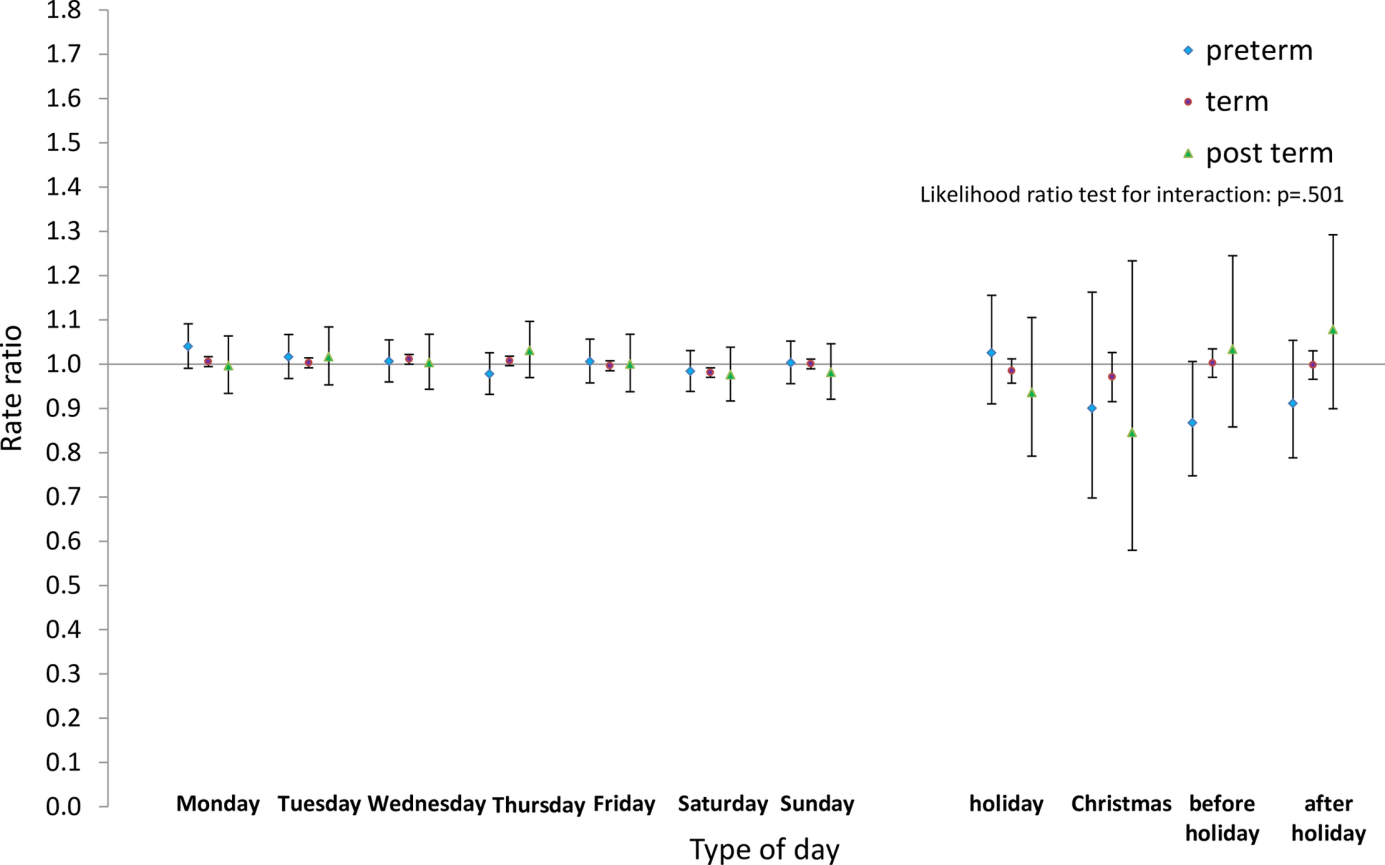
Rate ratios and confidence intervals for type of day differences by gestational age: Spontaneous onset, instrumental birth. Vertical axes show rate ratios relative to the overall average for each gestational age group within each figure. Error bars show 99% confidence intervals. Likelihood Ratio Test of *H*_0_: Type of day differences do not differ between term, pre-term and post-term births.

**Fig 11:**
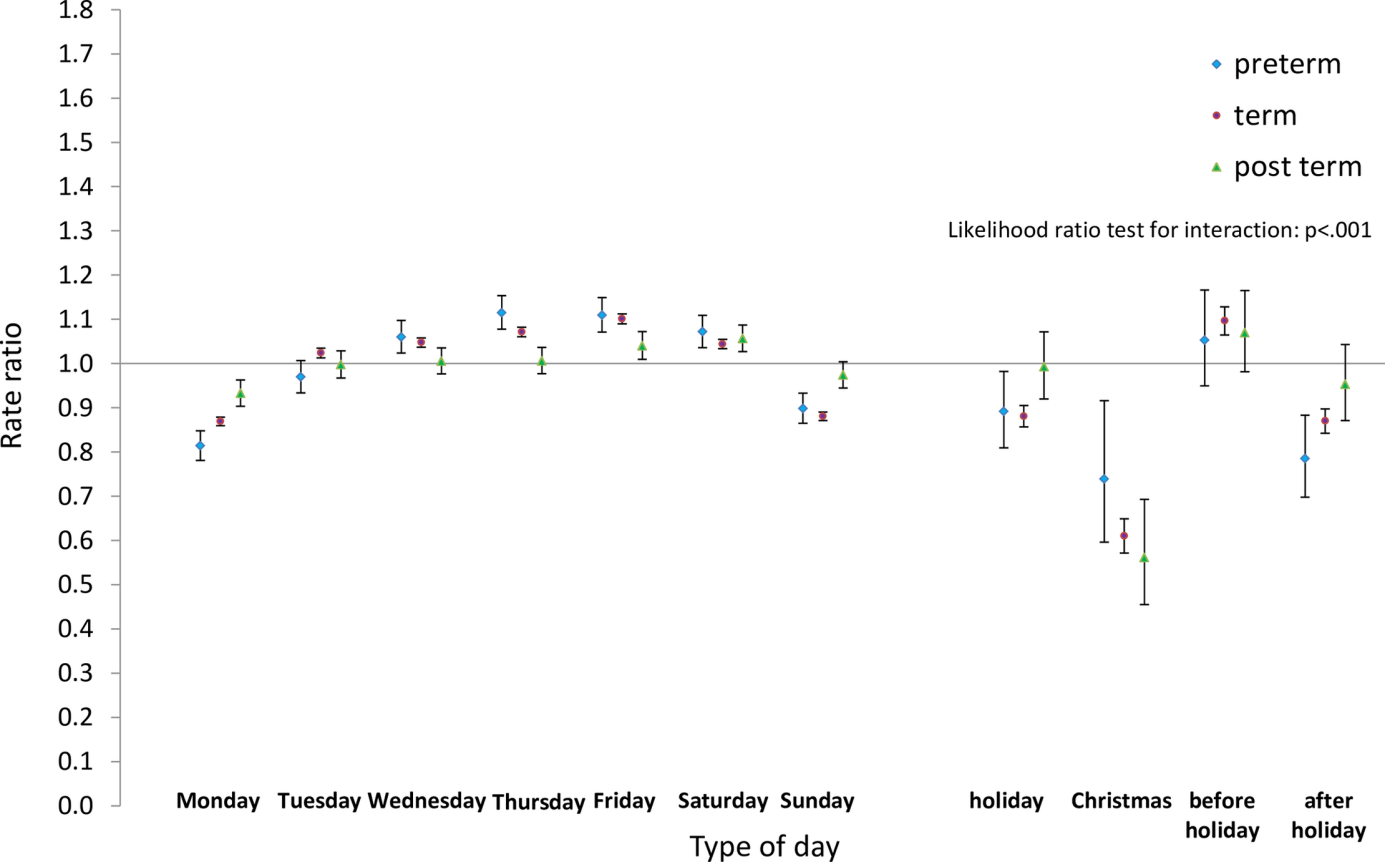
Rate ratios and confidence intervals for type of day differences by gestational age: Induced onset, spontaneous birth. Vertical axes show rate ratios relative to the overall average for each gestational age group within each figure. Error bars show 99% confidence intervals. Likelihood Ratio Test of *H*_0_: Type of day differences do not differ between term, pre-term and post-term births.

**Fig 12:**
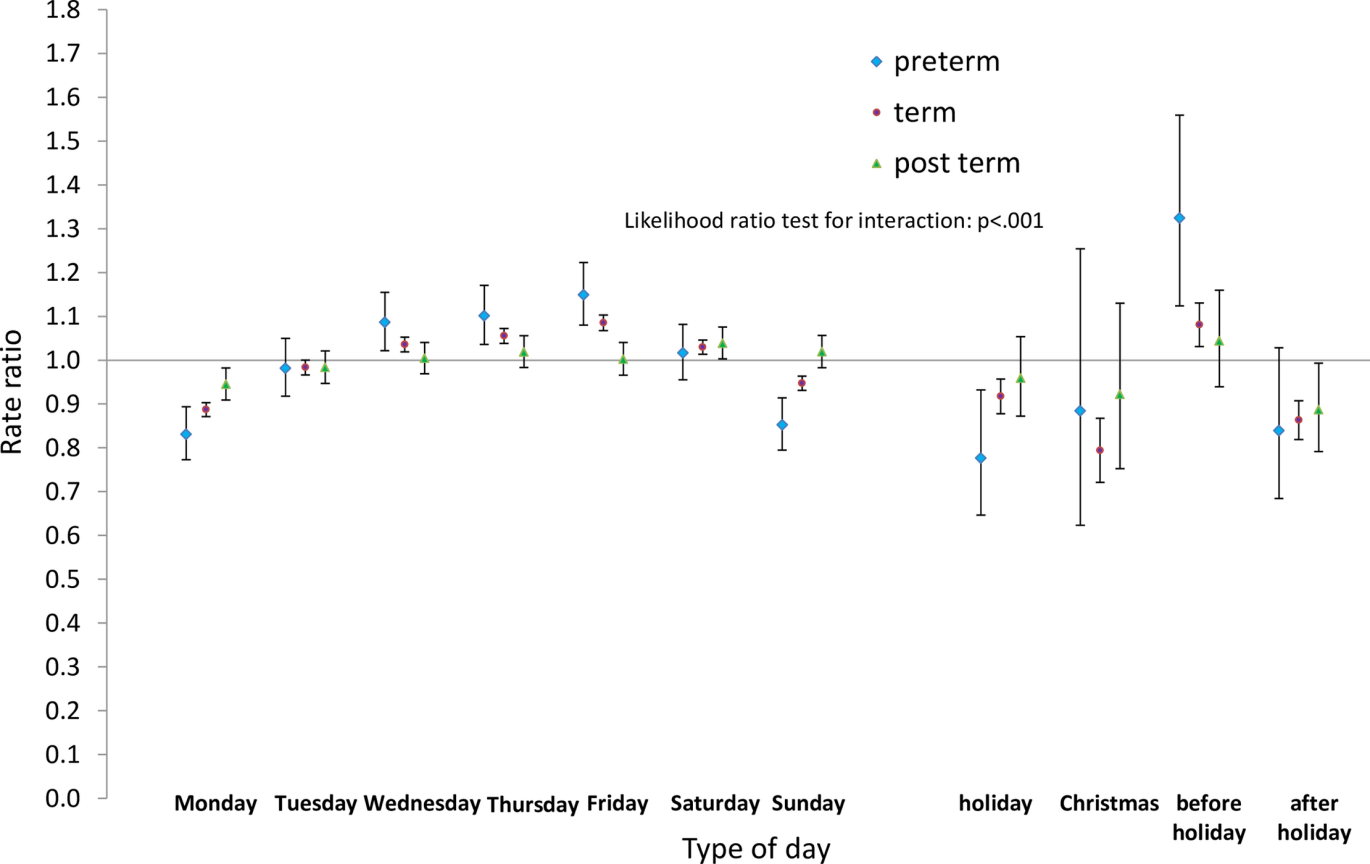
Rate ratios and confidence intervals for type of day differences by gestational age: Induced onset, emergency caesarean. Vertical axes show rate ratios relative to the overall average for each gestational age group within each figure. Error bars show 99% confidence intervals. Likelihood Ratio Test of *H*_0_: Type of day differences do not differ between term, pre-term and post-term births.

**Fig 13:**
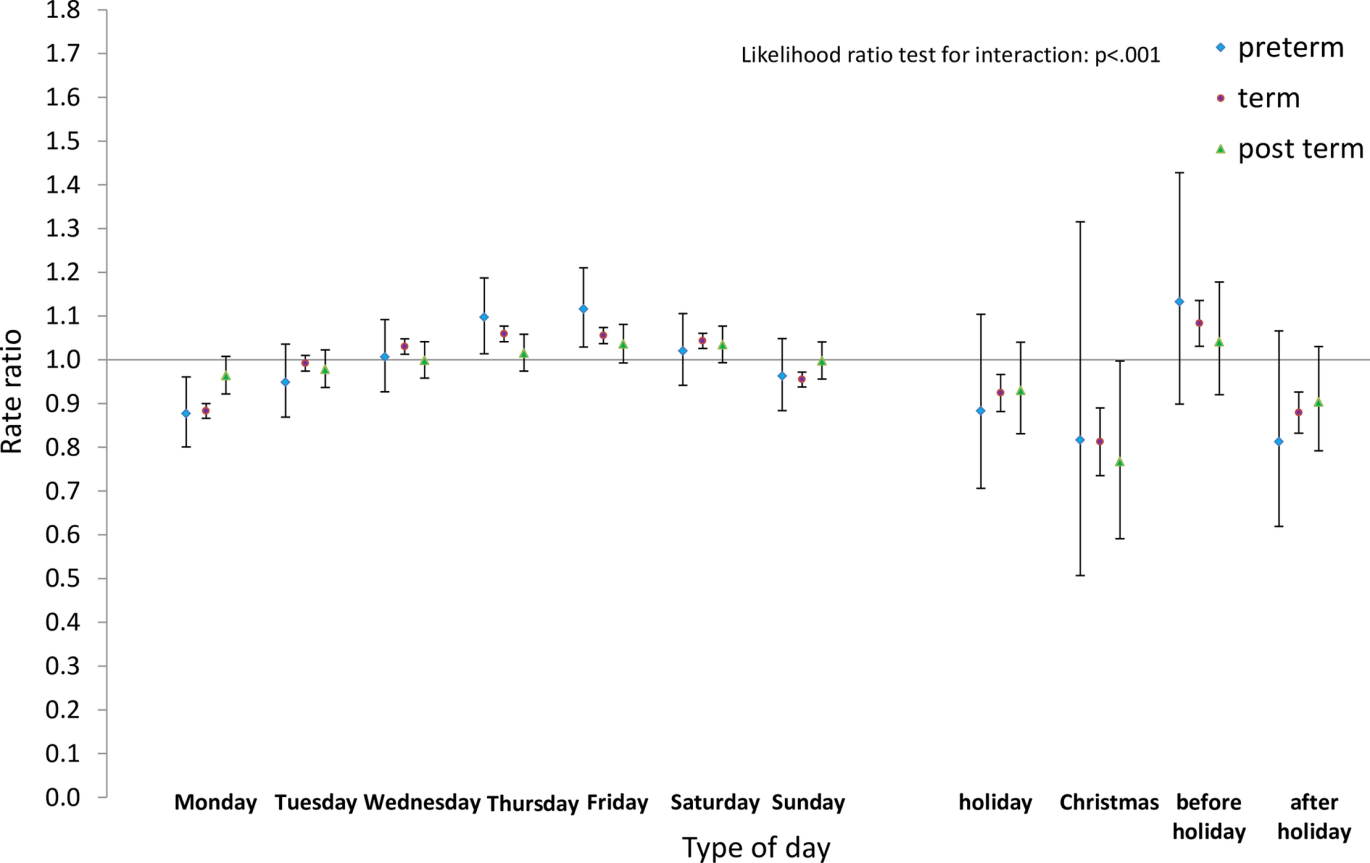
Rate ratios and confidence intervals for type of day differences by gestational age: Induced onset, instrumental birth. Vertical axes show rate ratios relative to the overall average for each gestational age group within each figure. Error bars show 99% confidence intervals. Likelihood Ratio Test of *H*_0_: Type of day differences do not differ between term, pre-term and post-term births.

**Fig 14:**
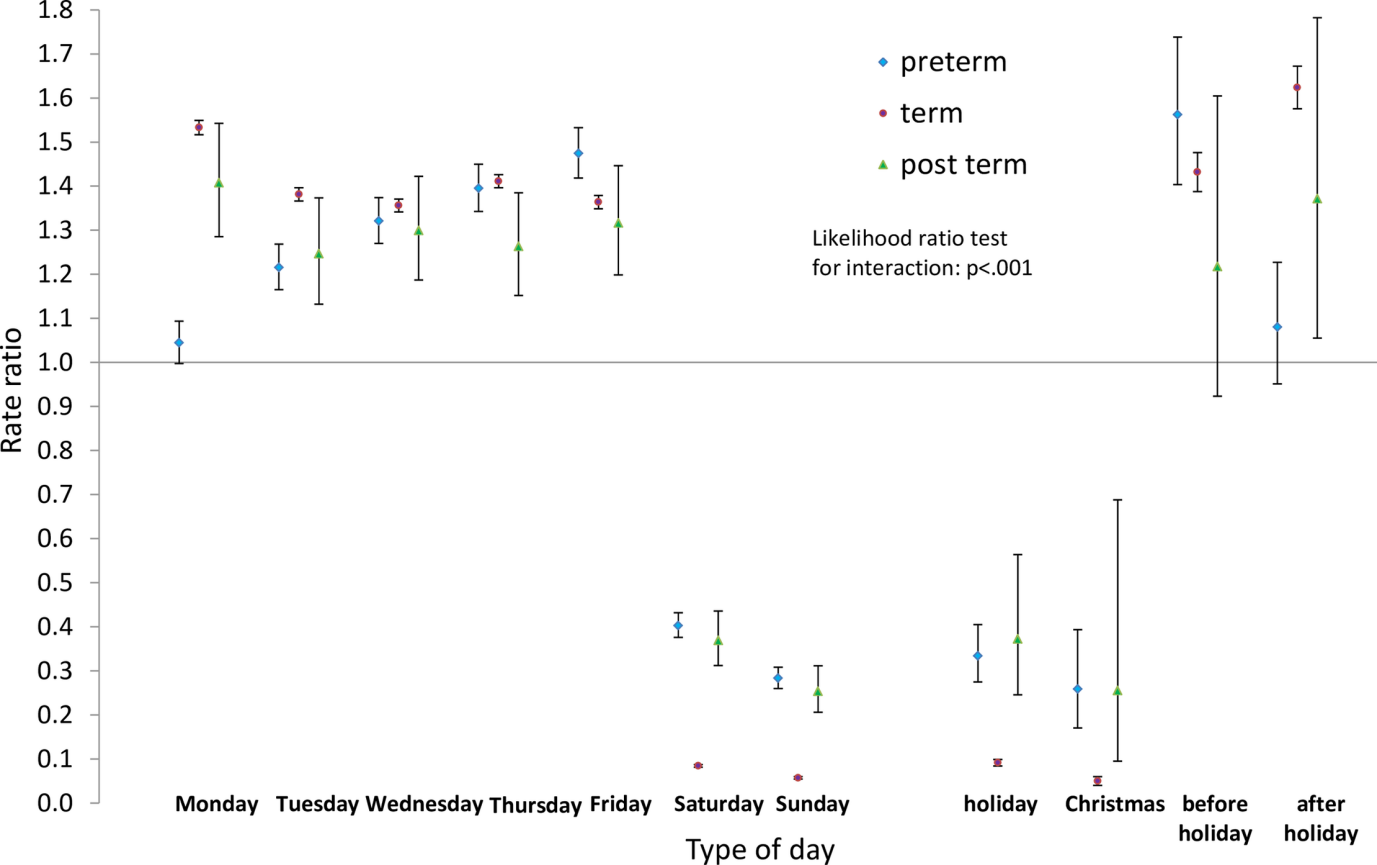
Rate ratios and confidence intervals for type of day differences by gestational age: Elective caesarean. Vertical axes show rate ratios relative to the overall average for each gestational age group within each figure. Error bars show 99% confidence intervals. Likelihood Ratio Test of *H*_0_: Type of day differences do not differ between term, pre-term and post-term births.

**Fig 15:**
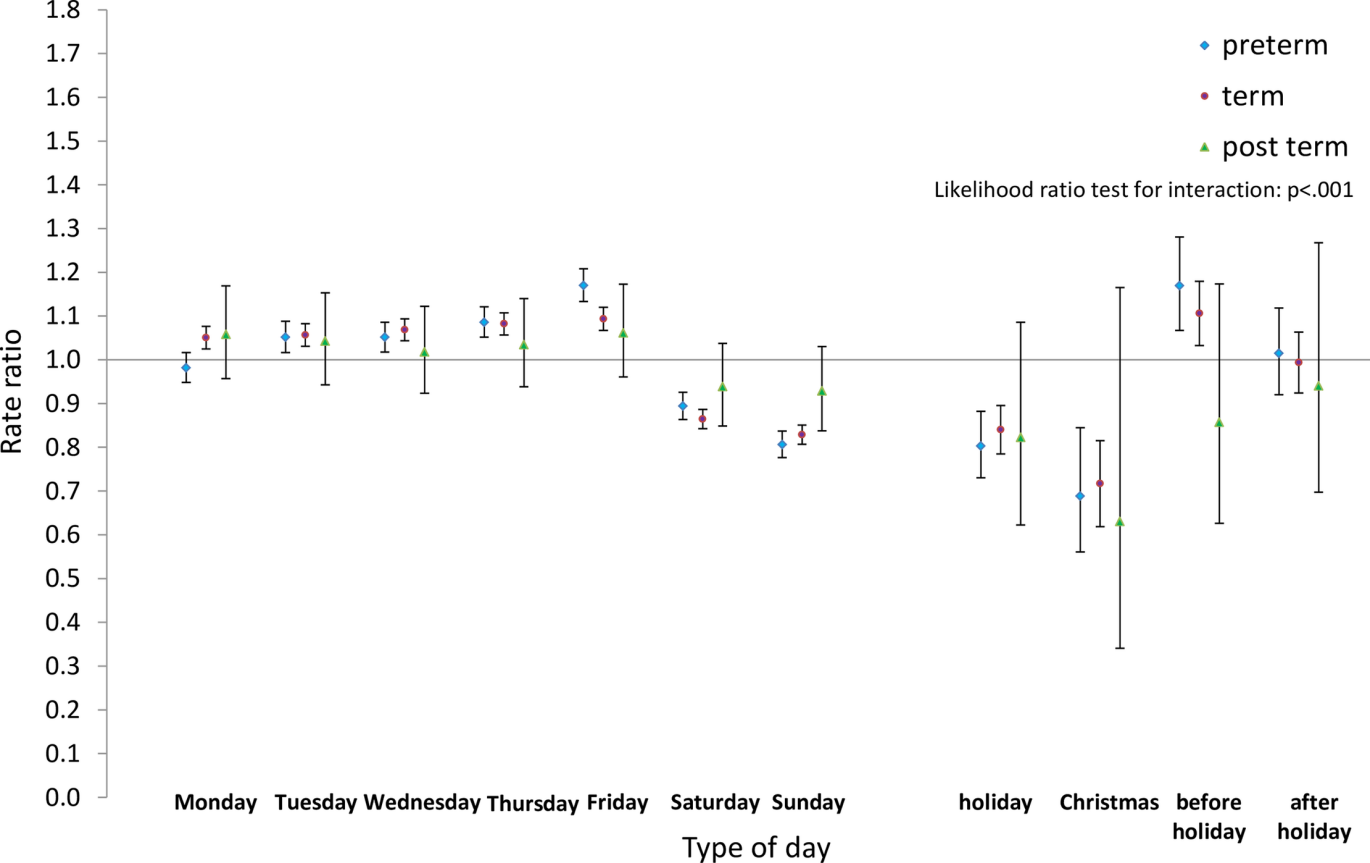
Rate ratios and confidence intervals for type of day differences by gestational age: Emergency caesarean, no labour. Vertical axes show rate ratios relative to the overall average for each gestational age group within each figure. Error bars show 99% confidence intervals. Likelihood Ratio Test of *H*_0_: Type of day differences do not differ between term, pre-term and post-term births.

Next we analysed the differences by type of day within each group for pre-term and post-term births as well as for term births. Estimated rate ratios and their confidence intervals, and p-values relating to the interaction effect for all eight groups, are shown in Figs 8–15. Our analysis suggests that gestational age moderated differences by type of day in six of the eight groups (that is, differences by type of day were not the same for all gestational age groups). The exceptions were emergency caesareans and instrumental births after spontaneous onset. For these, the interaction between gestational age and ‘type of day’ in predicting number of births per day was not statistically significant (see Figs 9 and 10).

For spontaneous births after spontaneous onset, shown in Fig 8, there was evidence of differences by type of day for term births, as reported above. On the other hand, there was no evidence of differences by type of day among pre-term births. As all the confidence intervals for post-term births were wider than and included the intervals for term births, no statistically reliable differences could be detected between these two groups.

Among induced births (Figs 11–13), the average numbers of births by day follow the same overall pattern regardless of gestational age group, but differences between types of days tend to be widest among pre-term births, and narrowest among post-term births.

For elective caesareans (Fig 14), the pattern among post-term births is similar to that among term births, although differences between types of days are somewhat smaller for post-term births. Pre-term elective caesareans, follow a different pattern, however, with numbers being highest on Fridays and on weekdays before a holiday, and lower on Mondays and weekdays after a holiday than on other weekdays.

Finally, in emergency caesareans without prior onset of labour, again there is little to suggest differences between post-term and term births (Fig 15). Pre-term births in this group follow a similar pattern to pre-term elective caesareans, however, with numbers being highest on Fridays and on days before holidays.

## Discussion

This is the first study to analyse, at a national level for England, data showing how the numbers of births vary by time of day and how these patterns vary by onset of labour and mode birth and from day to day. Having a large dataset with over five million births over a ten year period made it possible analyse the effects of time of day and day of the week, and produce estimates with good precision, even for the less common combinations of onset and birth.

### Hour of birth

This study showed that spontaneous births after spontaneous onset showed broadly the same sinusoidal patterns, peaking at night, seen in local analyses in England in 1950 and 1951 [25,42] Births at home in England and Wales from 2005 to 2014, which we have analysed separately, also show similar patterns as did home births in Birmingham in 1950 and 1951 [25]. Similar patterns were also visible among spontaneous births in a hospital in Spain in 1991, 2005 and 2007 [30].

In contrast, while data about spontaneous births in New York State in 1929 and 1936 showed these same patterns, numbers of spontaneous births in hospitals in the United States in 2013 were higher between noon and 5pm than at other times, but the reasons for this were not analysed. The older patterns were still present in out-of-hospital births in the United States, which showed a pattern similar to that in earlier, with numbers of births being highest between 3am and 5am [31].

While patterns of spontaneous births have remained unchanged in England, overall patterns of birth have changed with the rise in rates of obstetric intervention. The rising rates of elective caesarean section have concentrated increasing numbers of births onto weekday mornings. In contrast, although the rationale for increasing rates of induction in the 1970s was to concentrate births into day time hours, our data show that numbers of induced births peak at night, irrespective of the mode of birth. Overall, 48.1 per cent of births in our study data took place in the 12 hours from 6pm to 6am, compared with 39.5 per cent in the US in 2013 (Curtin S, personal communication).

The shapes of the distributions also differed between the UK and the US. In the US, elective caesareans were concentrated into mornings with two peaks at 8am and 12 noon, while numbers of emergency caesareans were highest during late afternoons and early evenings. Numbers of induced vaginal births showed a very different pattern from those in England, being highest between 12 noon and 6pm. Vaginal births after spontaneous onset varied considerably less but numbers were higher between noon and 5pm than at other times. These patterns illustrate the obstetric practice and use of interventions in the US.

A study in the Netherlands using data from 1990 suggested the type of care provider may also affect the time of birth. Women without obstetric intervention who had care from midwives had shorter labours than those who had care from obstetricians [32]. Among women receiving care from midwives, numbers of births to primiparous women peaked between 8am and 9am and births to multiparous women peaked at 5am. For women cared for by obstetricians, the corresponding peaks were over five hours later, between 2pm and 3pm for primiparous women and between 8am and 9am for multiparous women. Although clinical factors may have played a part in selection and the duration of labour, these comparisons raise questions about differences in styles of practice and settings for care. It is notable that in the US, the proportion of women giving birth in hospital who have midwifery care is low, while in England, midwives are the lead carer for most women who do not have obstetric intervention.

### Day of birth

The overall weekly patterns of births in our study are similar to those seen in analyses of registered births in England and Wales by day from 1970–76 [7,43] and from 1979–96 [8], which showed pronounced weekly cycles and lower numbers of births on public holidays, particularly in NHS hospitals with consultant units. The marked day of the week differences in patterns of birth after induced labour or by elective caesarean were also apparent in tabulations of Hospital In-Patient Enquiry (HIPE) data for 1980 [18] and in tables published in reports of Maternity HES for years from 1994–95 to 2005–06 [19,44].

These patterns have also been seen in many other countries and the consensus is that they reflect staff working patterns, especially the concentration of elective caesareans onto weekday mornings. In contrast, emergency caesareans would be expected in response to an urgent clinical need and thus be independent of the day of the week. Yet we found that numbers of emergency caesareans without any record of prior onset of labour were considerably lower on Sundays and holidays than on weekdays, and that their numbers were highest on days immediately preceding weekends and public holidays. Coding discrepancies may have contributed to our findings relating to emergency and elective caesareans. We found that a small number of births coded as elective caesarean sections occurred at weekends or on holidays. Some of these may have been wrongly coded. Coding discrepancies in mode of births have been found to be more common for elective and emergency caesarean sections than for other modes of birth [45].

It is also difficult to find a ready explanation for the finding that numbers of spontaneous births after spontaneous onset varied by days of the week, although to a much lesser extent. Over the years in our study, average numbers of these births were about one per cent lower on Saturdays, two per cent lower on Sundays and public holidays apart from Christmas, and seven per cent lower on Christmas and Boxing Day, compared to the overall daily average. It was not surprising, however, as similar patterns were seen in past analyses of births without obstetric intervention. Among births occurring at home or in NHS hospitals without a consultant obstetric unit from 1970–76, numbers of births were lower at weekends [7,17] and all the differences widened as induction rates rose from 1970 to 1976 [6]. They were also apparent in the HIPE and HES data referred to above.

A Lancet editorial commenting on the earlier data and pointing to the higher proportions of low birthweight babies among those born at weekends, suggested that this might reflect selective induction of term babies during the week [9,46] and prompted correspondence suggesting possible associations with variations in stress levels [16,47,48].

### Preterm births

Unlike the earlier analyses, we had data available to analyse preterm, term and post term births separately. We found that, unlike those at term, numbers of spontaneous preterm births after spontaneous onset did not vary by time of day and were not lower at weekends and on holidays. A similar result was found in a study of 2,005,096 births from 1 January 1960 to 30 September 1994 in Denmark. Lower than usual numbers of term births occurred on Christmas Eve, when the most important family gathering takes place, and the following three days, but there was no reduction in numbers of preterm births [49].

### Strengths and limitations

These analyses were based on a large dataset, derived from over five million births, giving sufficient numbers of births for detailed analyses. By linking three datasets together, we have greatly increased the numbers of variables available for analysis and there is scope for further analysis to look at social, demographic and clinical factors which we have not touched on here. Linkage has also made it possible to use the much more complete data about gestational age recorded at birth notification to compensate for the poor quality data about gestational age recorded in HES.

On the other hand, we had to exclude 15.9 per cent of the births in our study dataset from analysis, mainly because data about onset of labour was missing. This could mean that the ‘maternity tail’ was missing from HES records for a number of maternity units as well as for some individual births. This has been a long standing problem [50], but the situation improved considerably over the years 2003 to 2010 [38]. Unfortunately the situation began to deteriorate at the end of our study period and in subsequent years [51]. In addition, there are questions about the quality of data in HES submitted by some maternity units, but we could not directly evaluate the recording quality of key variables, particularly gestational age, onset of labour, and mode of birth. Overall, it is thus possible that some of our estimates are influenced by missing or inaccurate information.

Even after using data linkage, some key variables used in previous analyses were not available to us. Previous analyses showed differences by parity in the timing of birth, but parity is missing from many HES records and until 2012 was not recorded at birth registration for births outside marriage. As part of our project, methods have been developed for imputing parity [52], so it could be used in future analyses. In addition, the time of onset of labour is not recorded in any of the datasets we have linked, perhaps because it is often difficult to obtain an accurate measure of the time when labour starts.[25]

### Questions for further research

These analyses pose questions for further research. Further analyses of this linked dataset could explore how differences in timing of birth vary between social and demographic groups and between maternity units, with their widely varying rates of obstetric intervention. Intervention rates for migrant women often differ from those of the host population, and these may influence the pattern of the timing of birth [53–55] Other questions, such as the psycho-social factors which can impact on the onset and duration of labour, and their implications for the provision of maternity care, need to be approached by other means.

### Implications for maternity care

Elective caesareans can be scheduled to fit day-time hours and the shift patterns of many hospital staff. On the other hand, over 70 per cent of all births in NHS maternity units in England occur either at weekends or on weekdays between 17:00 and 8:59, and thus outside of usual working hours. The peak time for births after induced labour is the hour before midnight, regardless of mode of birth, while for spontaneous births after spontaneous onset, it is around 4:00am. Maternity services in England continue to do a substantial proportion of their work out of hours. These patterns must be taken into account in decisions about midwifery and medical staffing, as well as in analyses of weekend or ‘out of hours’ effects in maternity care.

## Supporting information

S1 Appendix Coding of onset of labour and mode of birth. (DOCX)

S2 Appendix Checking for linkage bias. (DOCX)

S3 Appendix Checking for bias: Comparisons between all eligible births and study dataset. (DOCX)

S4 Appendix The average number of births per hour on non-holiday Thursdays. (DOCX)
