# Supplementary material for: Timing of singleton births by onset of labour and mode of birth in NHS maternity units in England, 2005–2014: A study of linked birth registration, birth notification, and hospital episode data
Source: PLoS One. 2018 Jun 14;13(6):e0198183. doi: 10.1371/journal.pone.0198183 (PMC6002087; doi:10.1371/journal.pone.0198183)
Supplement: S1 Appendix — (DOCX) [file pone.0198183.s001.docx]

**S1. Supplementary information about coding of onset of labour and mode of birth**

**Structure of records in Maternity Hospital Statistics (HES)**

Records of delivery episodes in the Maternity Hospital Episode Statistics should have information about the birth coded in the ‘maternity tail’ appended to the standard admitted patient care record, which includes codes for diagnoses according to the International Classification of Diseases, as well as codes for procedures carried out during the episode, using the OPCS-4 Classification of Interventions and Procedures. In some cases, the maternity tail is missing for whole maternity units and in others for individuals.

**Onset of labour**

Information about the onset of labour is included in the variable DELONSET in the HES maternity tail and coded as shown in Table S1.1.

**Table S1.1: Coding of Onset of Labour**

| **Variable DELONSET in HES maternity tail** | **Onset of Labour** |
| --- | --- |
| 1 = Spontaneous: the onset of regular contractions whether or not preceded by spontaneous rupture of the membranes | Spontaneous |
| 2 = Any caesarean section carried out immediately following the onset of labour, when the decision was made before labour | Caesarean |
| 3 = Surgical induction by amniotomy | Induced |
| 4 = Medical induction, including the administration of agents either orally, intravenously or intravaginally with the intention of initiating labour |  |
| 5 = Combination of surgical induction and medical induction |  |
| 8 = Not applicable (from 1996-97 onwards) | Missing |
| 9 = Not known: validation error |  |

**Mode of birth**

Coding of Mode of birth is illustrated in Table S1.2. Mode of birth is recorded in the variable delmeth-1 in the HES maternity tail. OPCS-4 codes for any procedures undertaken are included in the core HES record and are available for most births. Since the mid-1990s, the procedure codes have been used for mode of birth in routine Maternity HES publications[1].

Mode of birth was therefore derived using the OPCS-4 procedure codes which were recorded in the core record for 98 % of births. There were however some records with more than one delivery code. These could have arisen because of a multiple birth, failed forceps or erroneous data entry. In these cases, the most invasive mode was chosen as the mode of birth in the order caesarean, instrumental and spontaneous vaginal. For records where the mode of birth was coded as 9 or X to denote it was missing the information recorded in delmeth_1 in the maternity tail was used. For the 0.1 % of births, where no mode of birth was recorded in either place but there were several episode records for the same delivery, the record with the mode of birth coded in the range 0-8 was used. For records where the mode of birth was elective caesarean, we grouped 445,453 births where the onset was recorded as “caesarean” with 24,450 births where the onset was recorded as spontaneous, since exploratory analysis suggested that there was no difference between these groups of births with respect to time and day of birth.

**Table S1.2. Derivation of mode of birth using procedure codes in the HES delivery record, England**

| **Code** | **Categories used in delmeth-1 in HES maternity tail** | **OPCS-4 Code** | **Mode of birth** |
| --- | --- | --- | --- |
| 0 | Spontaneous vertex (normal vaginal delivery, occipitoanterior) | R24* | Spontaneous |
| 1 | Spontaneous other cephalic (cephalic vaginal delivery with abnormal presentation of head at delivery, without instruments, with or without manipulation) | R23* |  |
| 2 | Low forceps, not breech, including forceps delivery not otherwise specified (forceps, low application, without manipulation) | R215 or R218 or R219 | Instrumental |
| 3 | Other forceps, not breech, including high forceps and mid forceps (forceps with manipulation) | R211 or R212 or R213 or R214 |  |
| 4 | Ventouse, vacuum extraction | R22* |  |
| 5 | Breech, including partial breech extraction (spontaneous delivery assisted or unspecified) | R20* | Spontaneous |
| 6 | Breech | R19* | Instrumental |
| 7 | Elective caesarean section | R17* | Elective Caesarean |
| 8 | Emergency caesarean section | R18* | Emergency Caesarean (and other surgical) |
| 9 | Other | R25* | Missing |
| X | Not known | Otherwise |  |

*Note: For further explanation of OPCS-4 codes, please consult the explanatory notes provided by the Health and Social Care Information Centre [2].*

**Bibliography**

1 Department of Health. NHS maternity statistics, England: 1989-90 to 1994-95. London: 1997.

2 The Health and Social Care Information Centre. NHS Maternity Statistics: explanatory notes. 2011. https://digital.nhs.uk/catalogue/PUB03071
