# Supplementary material for: Timing of singleton births by onset of labour and mode of birth in NHS maternity units in England, 2005–2014: A study of linked birth registration, birth notification, and hospital episode data
Source: PLoS One. 2018 Jun 14;13(6):e0198183. doi: 10.1371/journal.pone.0198183 (PMC6002087; doi:10.1371/journal.pone.0198183)
Supplement: S2 Appendix — (DOCX) [file pone.0198183.s002.docx]

**Supplement S2: Checking for linkage bias**

Tables S2.1 to S2.8 show distributions of key demographic and time-of-birth variables for three sets of births defined as follows:

| **Category** | **Definition** | **Number of records** |
| --- | --- | --- |
| ONS birth records | All registered births linked to notified births | 6,468,586 |
| Linked and matched births | ONS birth records included in the study | 6,137,945 |
| Unlinked or unmatched births | ONS births excluded from the study because they were unlinked or unmatched | 330,641 |

Chi-square tests were conducted to test the null hypothesis that the distribution of a demographic variable is the same in the “linked and matched births” as in the “Unlinked or Unmatched births”.

**Table S2.1: Comparisons between percentage distributions of ONS birth records and linked and matched births by year of birth**

| Year of birth | Linked and matched births | Unlinked or  unmatched | All ONS birth records |
| --- | --- | --- | --- |
| 2005 | 9.03 | 13.55 | 9.26 |
| 2006 | 9.35 | 14.24 | 9.60 |
| 2007 | 9.70 | 13.16 | 9.88 |
| 2008 | 10.12 | 10.66 | 10.14 |
| 2009 | 10.12 | 9.66 | 10.10 |
| 2010 | 10.45 | 7.39 | 10.29 |
| 2011 | 10.46 | 7.36 | 10.30 |
| 2012 | 10.57 | 8.44 | 10.46 |
| 2013 | 10.15 | 7.48 | 10.01 |
| 2014 | 10.06 | 8.05 | 9.95 |
| All | 100.00 | 100.00 | 100.00 |
| Total number | 6,137,945 | 330,641 | 6,468,586 |

Note: χ^2^ test (H_0_: distribution of linked and matched births is the same as the distribution of unlinked or unmatched births): p < .001 (χ^2^ = 29363.2, df = 9)

**Table S2.2: Comparisons between percentage distributions of ONS birth records and linked and matched births by month of birth**

| Month of birth | Linked and matched births | Unlinked or unmatched | All ONS birth records |
| --- | --- | --- | --- |
| January | 8.26 | 8.55 | 8.28 |
| February | 7.56 | 8.21 | 7.59 |
| March | 8.21 | 9.75 | 8.29 |
| April | 8.00 | 7.85 | 8.00 |
| May | 8.45 | 8.11 | 8.43 |
| June | 8.28 | 7.97 | 8.27 |
| July | 8.70 | 8.37 | 8.68 |
| August | 8.62 | 8.18 | 8.60 |
| September | 8.68 | 8.10 | 8.65 |
| October | 8.69 | 8.27 | 8.67 |
| November | 8.22 | 8.08 | 8.21 |
| December | 8.31 | 8.55 | 8.32 |
| All | 100.00 | 100.00 | 100.00 |
| Total number | 6,137,945 | 330,641 | 6,468,586 |

Note: χ^2^ test (H_0_: distribution of linked and matched births is the same as the distribution of unlinked or unmatched births): p < .001 (χ^2^ = 1513.1, df = 11)

**Table S2.3: Comparisons between percentage distributions of ONS birth records linked and matched births by region of residence**

| Region of residence | Linked and matched births | Unlinked or unmatched | All ONS birth records |
| --- | --- | --- | --- |
| East Midlands | 7.39 | 3.71 | 7.20 |
| East of England | 9.75 | 8.56 | 9.69 |
| London | 19.30 | 27.27 | 19.71 |
| North East | 4.73 | 2.24 | 4.61 |
| North West | 13.32 | 7.44 | 13.02 |
| South Central | 7.49 | 3.96 | 7.31 |
| South East Coast | 7.74 | 4.97 | 7.60 |
| South West | 8.06 | 3.50 | 7.82 |
| West Midlands | 10.93 | 5.41 | 10.64 |
| Yorkshire/Humber | 9.97 | 4.51 | 9.69 |
| Home | 1.23 | 27.05 | 2.55 |
| Elsewhere | 0.11 | 1.39 | 0.17 |
| All | 100.00 | 100.00 | 100.00 |
| Total number | 6,137,943 | 330,638 | 6,468,581 |

Note: 5 births had an unknown birth region and were excluded from this table. Note: χ^2^ test (H_0_: distribution of linked and matched births is the same as the distribution of unlinked or unmatched births): p < .001 (χ^2^ = 915877.2, df = 11)

**Table S2.4: Comparisons between percentage distributions of ONS birth records with linked and matched births: gestational age group**

| Gestational Age Group | Linked and matched births | Not linked or not matched | All ONS birth records |
| --- | --- | --- | --- |
| Missing or below 22 weeks | 0.76 | 1.32 | 0.79 |
| Pre-term | 6.05 | 6.73 | 6.08 |
| Term | 89.19 | 88.71 | 89.17 |
| Post-term | 4.00 | 3.23 | 3.96 |
| All | 100.00 | 100.00 | 100.00 |
| Total number | 6,137,945 | 330,641 | 6,468,586 |

Note: χ^2^ test (H_0_: distribution of linked and matched births is the same as the distribution of unlinked or unmatched births): p < .001 (χ^2^ = 1947.9, df = 3)

**Table S2.5: Comparisons between percentage distributions of ONS birth records with linked and matched births: mother’s age**

| Mother’s age | Linked and matched births | Unlinked or unmatched | All ONS birth records |
| --- | --- | --- | --- |
| Under 15 | 0.03 | 0.03 | 0.03 |
| 15-19 | 5.58 | 4.98 | 5.55 |
| 20-24 | 18.59 | 16.41 | 18.48 |
| 25-29 | 27.42 | 24.89 | 27.29 |
| 30-34 | 28.69 | 29.27 | 28.72 |
| 35-39 | 15.96 | 19.15 | 16.13 |
| 40-44 | 3.54 | 4.81 | 3.6 |
| 45 or older | 0.19 | 0.47 | 0.2 |
| All | 100.00 | 100.00 | 100.00 |
| Total number | 330,639 | 6,137,945 | 6,468,584 |

Note: Two unlinked records had no information about mother’s age. χ^2^ test (H_0_: distribution of linked and matched births is the same as the distribution of unlinked or unmatched births): p < .001 (χ^2^ = 6433.4, df = 7)

**Table S2.6: Comparisons between percentage distributions of ONS birth records with linked and matched births: baby’s sex**

| Baby’s Sex | Linked and matched births | Unlinked or unmatched | All ONS birth records |
| --- | --- | --- | --- |
| Female | 48.66 | 49.20 | 48.69 |
| Male | 51.34 | 50.80 | 51.31 |
| All | 100.00 | 100.00 | 100.00 |
| Total number | 6,137,945 | 330,641 | 6,468,586 |

Note: Two unlinked records had no information about mother’s age. χ^2^ test (H_0_: distribution of linked and matched births is the same as the distribution of unlinked or unmatched births): p < .001 (χ^2^ = 36.09, df = 1)

**Table S2.7: Comparisons between percentage distributions of ONS birth records with linked and matched births: type of day of birth**

| Day of birth | Linked and matched births | Unlinked or unmatched | All ONS birth records |
| --- | --- | --- | --- |
| Monday | 13.05 | 13.00 | 13.05 |
| Tuesday | 13.33 | 13.25 | 13.32 |
| Wednesday | 14.59 | 14.59 | 14.59 |
| Thursday | 14.56 | 14.40 | 14.55 |
| Friday | 13.59 | 13.55 | 13.59 |
| Saturday | 12.94 | 13.32 | 12.96 |
| Sunday | 12.15 | 12.06 | 12.14 |
| Holiday | 1.93 | 1.98 | 1.93 |
| Christmas | 0.43 | 0.46 | 0.43 |
| Last weekday before a holiday period | 1.75 | 1.73 | 1.75 |
| First weekday after a holiday period | 1.69 | 1.66 | 1.68 |
| All | 100.00 | 100.00 | 100.00 |
| Total number | 6,137,945 | 330,641 | 6,468,586 |

Note: χ^2^ test (H_0_: distribution of linked and matched births is the same as the distribution of unlinked or unmatched births): p < .001 (χ^2^ = 58.9, df = 10)

**Table S2.8: Comparisons between percentage distributions of ONS birth records with linked and matched births: hour of birth**

|  | Linked and matched births | Unlinked or unmatched | All ONS birth records |
| --- | --- | --- | --- |
| 0:00 – 2:59 | 12.46 | 12.55 | 12.47 |
| 3:00 – 5:59 | 12.23 | 12.84 | 12.26 |
| 6:00 – 8:59 | 10.82 | 12.03 | 10.89 |
| 9:00 – 11:59 | 16.28 | 14.88 | 16.21 |
| 12:00 – 14:59 | 13.01 | 12.74 | 13.00 |
| 15:00 – 17:59 | 11.98 | 11.74 | 11.97 |
| 18:00 – 20:59 | 11.29 | 11.36 | 11.29 |
| 21:00 – 23:59 | 11.92 | 11.84 | 11.92 |
| All | 100.00 | 100.00 | 100.00 |
| Total number | 6,092,298 | 237,145 | 6,409,443 |

Note: 59,143 of all registered births had no information on the time of birth. χ^2^ test (H_0_: distribution of linked and matched births is the same as the distribution of unlinked or unmatched births): p < .001 (χ^2^ = 921.2, df = 7)
