# Supplementary material for: Timing of singleton births by onset of labour and mode of birth in NHS maternity units in England, 2005–2014: A study of linked birth registration, birth notification, and hospital episode data
Source: PLoS One. 2018 Jun 14;13(6):e0198183. doi: 10.1371/journal.pone.0198183 (PMC6002087; doi:10.1371/journal.pone.0198183)
Supplement: S3 Appendix — (DOCX) [file pone.0198183.s003.docx]

**Appendix S3: Checking for bias: comparisons between eligible births and study dataset**

Tables S3.1 to S3.9 show distributions of demographic and time-of-birth variables for three subsets of singleton births in England, 2005 – 2014 defined as follows

| Births in NHS maternity units | all singleton births in NHS maternity units | 6,054,536 |
| --- | --- | --- |
| Study dataset | subset of births in NHS maternity units, after excluding births with information about gestational age, onset of labour or mode of birth missing | 5,093,615 |
| Birth in NHS maternity units excluded from study dataset | Births in NHS maternity units excluded because information about gestational age, onset of labour or mode of birth was missing | 960,921 |

**Table S3.1: Comparisons between percentage distributions of all births in NHS maternity units to Study Data Set: birth year**

| Year of birth | Study dataset | Births in NHS Maternity units Excluded from study dataset | Births in NHS Maternity units |
| --- | --- | --- | --- |
| 2005 | 8.53 | 11.99 | 9.08 |
| 2006 | 8.69 | 13.05 | 9.38 |
| 2007 | 8.43 | 16.58 | 9.73 |
| 2008 | 9.63 | 12.68 | 10.11 |
| 2009 | 10.52 | 7.88 | 10.10 |
| 2010 | 10.98 | 7.44 | 10.42 |
| 2011 | 11.12 | 6.87 | 10.44 |
| 2012 | 11.26 | 6.80 | 10.55 |
| 2013 | 10.60 | 7.71 | 10.14 |
| 2014 | 10.24 | 8.98 | 10.04 |
| All | 100.00 | 100.00 | 100.00 |
| Total number | 5,093,615 | 960,921 | 6,054,536 |

Note: χ^2^ test (H_0_: distribution of births in study dataset is the same as the distribution of excluded births): p < .001 (χ^2^ = 142070, df = 9)

**Table S3.2: Comparisons between percentage distributions of all births in NHS maternity units to study dataset: birth month**

| Month of birth | Study dataset | Births in NHS Maternity units excluded from study dataset | Births in NHS Maternity units |
| --- | --- | --- | --- |
| January | 8.18 | 8.72 | 8.27 |
| February | 7.44 | 8.23 | 7.57 |
| March | 8.01 | 9.35 | 8.22 |
| April | 8.06 | 7.69 | 8.00 |
| May | 8.51 | 8.11 | 8.45 |
| June | 8.35 | 7.90 | 8.28 |
| July | 8.79 | 8.18 | 8.69 |
| August | 8.73 | 8.07 | 8.63 |
| September | 8.76 | 8.28 | 8.68 |
| October | 8.72 | 8.56 | 8.69 |
| November | 8.20 | 8.30 | 8.22 |
| December | 8.24 | 8.63 | 8.31 |
| All | 100.00 | 100.00 | 100.00 |
| Total number | 5,093,615 | 960,921 | 6,054,536 |

Note: χ^2^ test (H_0_: distribution of births in study dataset is the same as the distribution of excluded births): p < .001 (χ^2^ = 4343.6, df = 11).

**Table S3.3: Comparisons between percentage distributions of all births in NHS maternity units to study dataset: birth region**

| Region of birth | Study dataset | Births in NHS Maternity units excluded from study dataset | Births in NHS Maternity units |
| --- | --- | --- | --- |
| East Midlands | 8.31 | 3.15 | 7.49 |
| East of England | 10.43 | 6.98 | 9.88 |
| London | 18.98 | 22.64 | 19.56 |
| North East | 4.71 | 5.25 | 4.80 |
| North West | 14.51 | 8.10 | 13.49 |
| South Central | 7.64 | 7.31 | 7.59 |
| South East Coast | 6.92 | 12.76 | 7.84 |
| South West | 7.57 | 11.30 | 8.17 |
| West Midlands | 10.98 | 11.58 | 11.07 |
| Yorkshire/Humber | 9.95 | 10.94 | 10.11 |
| All | 100.00 | 100.00 | 100.00 |
| Total number | 5,093,615 | 960,921 | 6,054,536 |

Note: The categories ‘Home’, ‘Elsewhere’, and ‘Not known’ have been excluded from this table (they are excluded from the study by design). χ^2^ test (H_0_: distribution of births in study dataset is the same as the distribution of excluded births): p < .001 (χ^2^ = 119121, df = 9)

**Table S3.4: Comparisons between percentage distributions of all births in NHS maternity units to study dataset: gestational age group**

| Gestational age group | Study dataset | Births in NHS Maternity units excluded from study dataset | Births in NHS Maternity units |
| --- | --- | --- | --- |
| Pre-term | 5.86 | 7.67 | 6.14 |
| Term | 90.11 | 88.15 | 89.81 |
| Post-term | 4.03 | 4.19 | 4.05 |
| All | 100.00 | 100.00 | 100.00 |
| Total number | 5,093,615 | 914,676 | 6,008,291 |

Note: Births with unknown gestational age or gestational age below 22 weeks have been excluded from this table. χ^2^ test (H_0_: distribution of births in study dataset is the same as the distribution of excluded births): p < .001 (χ^2^ = 4503.2, df = 2).

**Table S3.5: Comparisons between percentage distributions of all births in NHS maternity units to study dataset: mother’s age**

| Mother’s age | Study dataset | Births in NHS Maternity units excluded from study dataset | Births in NHS Maternity units |
| --- | --- | --- | --- |
| Under 15 | 0.03 | 0.03 | 0.03 |
| 15-19 | 5.60 | 5.72 | 5.62 |
| 20-24 | 18.78 | 18.07 | 18.66 |
| 25-29 | 27.61 | 26.66 | 27.46 |
| 30-34 | 28.61 | 28.80 | 28.64 |
| 35-39 | 15.72 | 16.71 | 15.88 |
| 40-44 | 3.48 | 3.79 | 3.53 |
| 45 or older | 0.18 | 0.21 | 0.19 |
| All | 100.00 | 100.00 | 100.00 |
| Total number | 5,093,615 | 960,921 | 6,054,536 |

Note: χ^2^ test (H_0_: distribution of births in study dataset is the same as the distribution of excluded births): p < .001 (χ^2^ = 1269.7, df = 7).

**Table S3.6: Comparisons between percentage distributions of all births in NHS maternity units to study dataset: baby’s sex**

| Baby’s sex | Study dataset | Births in NHS Maternity units excluded from study dataset | Births in NHS Maternity units |
| --- | --- | --- | --- |
| Female | 48.64 | 48.64 | 48.64 |
| Male | 51.36 | 51.36 | 51.36 |
| All | 100.00 | 100.00 | 100.00 |
| Total number | 5,093,615 | 960,921 | 6,054,536 |

Note: χ^2^ test (H_0_: distribution of births in study dataset is the same as the distribution of excluded births): p = .996 (χ^2^ = 0.00003, df = 1).

**Table S3.7: Comparisons between percentage distributions of all births in NHS maternity units to study dataset: type of day of birth**

| Day of birth | Study dataset | Births in NHS Maternity units excluded from study dataset | Births in NHS Maternity units |
| --- | --- | --- | --- |
| Monday | 13.04 | 13.12 | 13.06 |
| Tuesday | 13.30 | 13.47 | 13.33 |
| Wednesday | 14.57 | 14.69 | 14.59 |
| Thursday | 14.55 | 14.68 | 14.57 |
| Friday | 13.57 | 13.76 | 13.60 |
| Saturday | 12.97 | 12.71 | 12.93 |
| Sunday | 12.20 | 11.77 | 12.13 |
| Holiday | 1.94 | 1.85 | 1.92 |
| Christmas | 0.43 | 0.44 | 0.43 |
| Last weekday before a holiday period | 1.74 | 1.80 | 1.75 |
| First weekday after a holiday period | 1.68 | 1.71 | 1.69 |
| All | 100.00 | 100.00 | 100.00 |
| Total number | 5,093,615 | 960,921 | 6,054,536 |

Note: χ^2^ test (H_0_: distribution of births in study dataset is the same as the distribution of excluded births): p < .001 (χ^2^ = 281.6, df = 10).

**Table S3.8: Comparisons between percentage distributions of all births in NHS maternity units to study dataset: hour of birth**

| Hour of birth | Study dataset | Births in NHS Maternity units excluded from study dataset | Births in NHS Maternity units |
| --- | --- | --- | --- |
| 0:00 – 2:59 | 12.51 | 12.08 | 12.44 |
| 3:00 – 5:59 | 12.26 | 11.72 | 12.17 |
| 6:00 – 8:59 | 10.83 | 10.43 | 10.77 |
| 9:00 – 11:59 | 16.17 | 17.19 | 16.34 |
| 12:00 – 14:59 | 12.96 | 13.54 | 13.05 |
| 15:00 – 17:59 | 11.95 | 12.31 | 12.01 |
| 18:00 – 20:59 | 11.33 | 11.10 | 11.30 |
| 21:00 – 23:59 | 11.98 | 11.63 | 11.93 |
| All | 100.00 | 100.00 | 100.00 |
| Total number | 5,048,369 | 951,563 | 5,999,932 |

Note: χ^2^ test (H_0_: distribution of births in study dataset is the same as the distribution of excluded births): p < .001 (χ^2^ = 1344.5, df = 7).

**Table S3.9: Comparisons between percentage distributions of all births in NHS maternity units to study dataset: mode of birth**

| Mode of birth | Study dataset | Births in NHS Maternity units excluded from study dataset | Births in NHS Maternity units |
| --- | --- | --- | --- |
| Spontaneous | 63.69 | 59.56 | 63.04 |
| Instrumental | 12.92 | 11.43 | 12.68 |
| Elective Caesarean | 9.23 | 12.59 | 9.76 |
| Emergency Caesarean | 14.16 | 16.41 | 14.52 |
| All | 100.00 | 100.00 | 100.00 |
| Total number | 5,093,615 | 949,879 | 6,043,494 |

Note: Births with unknown mode of birth have been excluded from this table. χ^2^ test (H_0_: distribution of births in study dataset is the same as the distribution of excluded births): p < .001 (χ^2^ = 15670, df = 3).
