# Supplementary material for: Timing of singleton births by onset of labour and mode of birth in NHS maternity units in England, 2005–2014: A study of linked birth registration, birth notification, and hospital episode data
Source: PLoS One. 2018 Jun 14;13(6):e0198183. doi: 10.1371/journal.pone.0198183 (PMC6002087; doi:10.1371/journal.pone.0198183)
Supplement: S4 Appendix — (DOCX) [file pone.0198183.s004.docx]

**Appendix S4: The average number of births per hour on non-holiday Thursdays**

Figures S4.1 to S4.8 show the distributions of mean number of births per hour on non-holiday Thursdays. This allows the reader to view in more detail the distributions shown in Figures 3-7 in the main text. Thursday was chosen as an example day.

**Figure S4.1: Average number of births per hour on a non-holiday Thursday in NHS maternity units in England, 2005 – 2014: Spontaneous onset and birth**


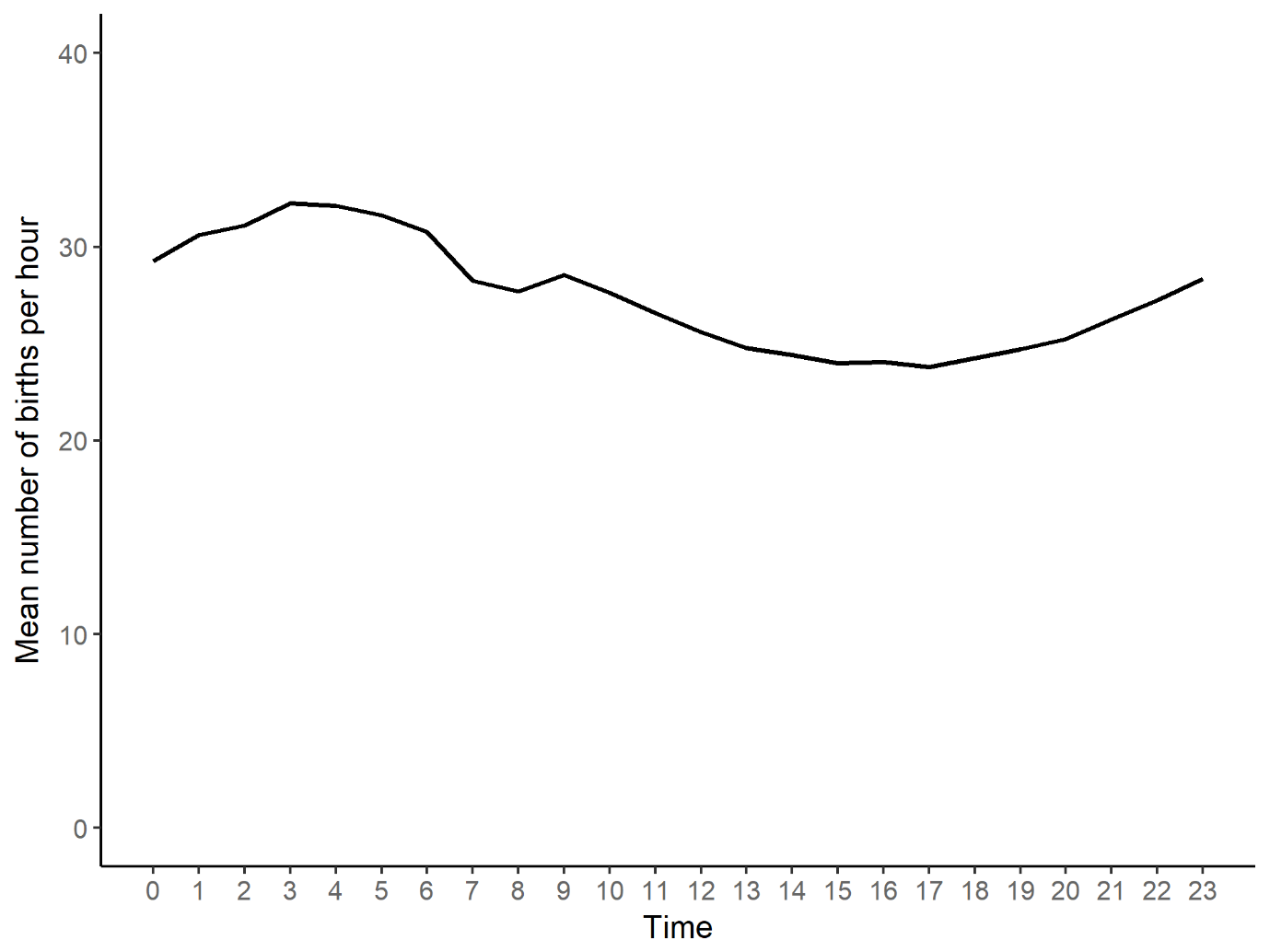


**Figure S4.2: Average number of births per hour on a non-holiday Thursday in NHS maternity units in England, 2005 – 2014: Spontaneous onset, instrumental birth**

**
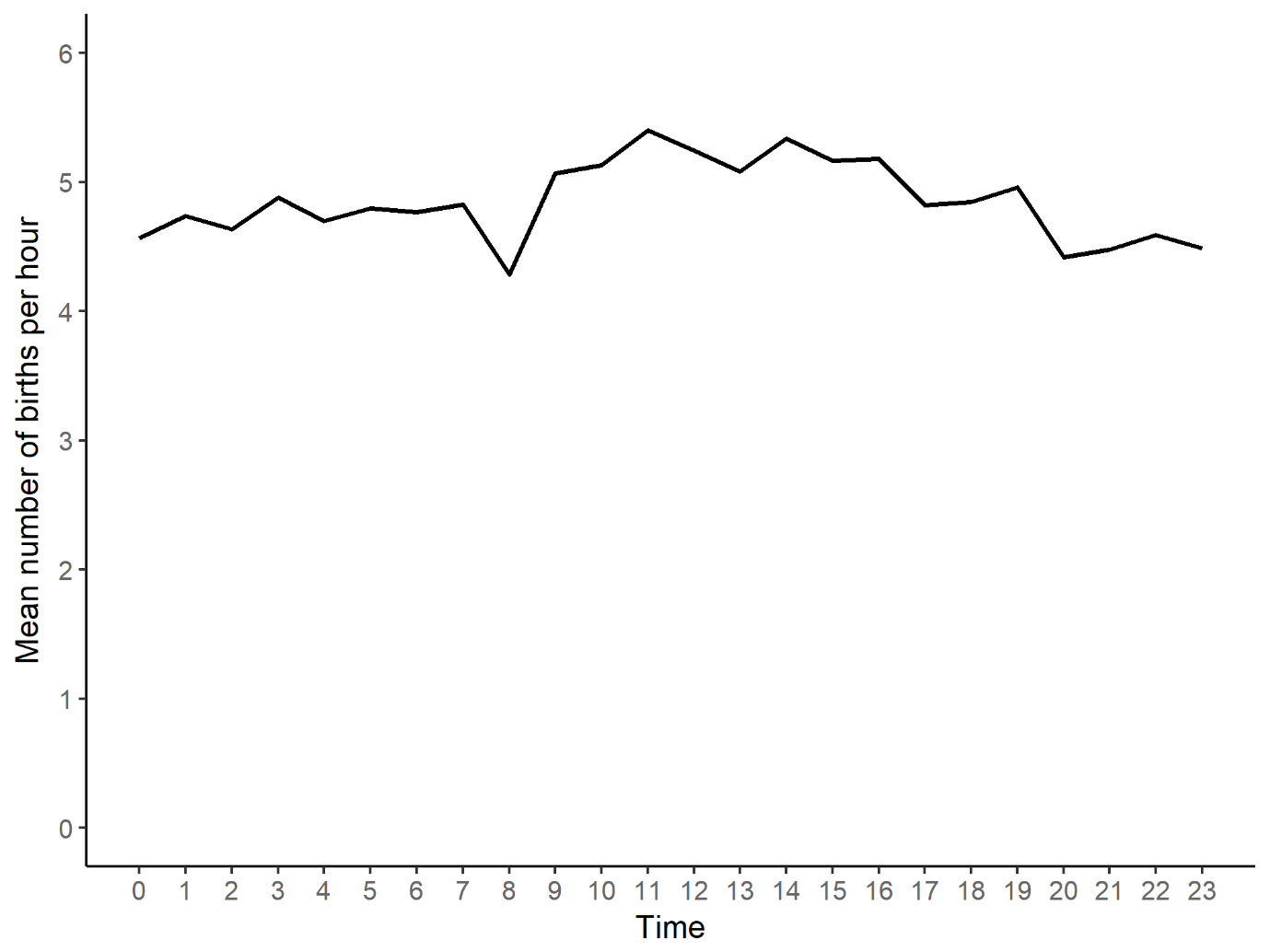
**

**Figure S4.3: Average number of births per hour on a non-holiday Thursday in NHS maternity units in England, 2005 – 2014: Spontaneous onset, emergency caesarean**

**
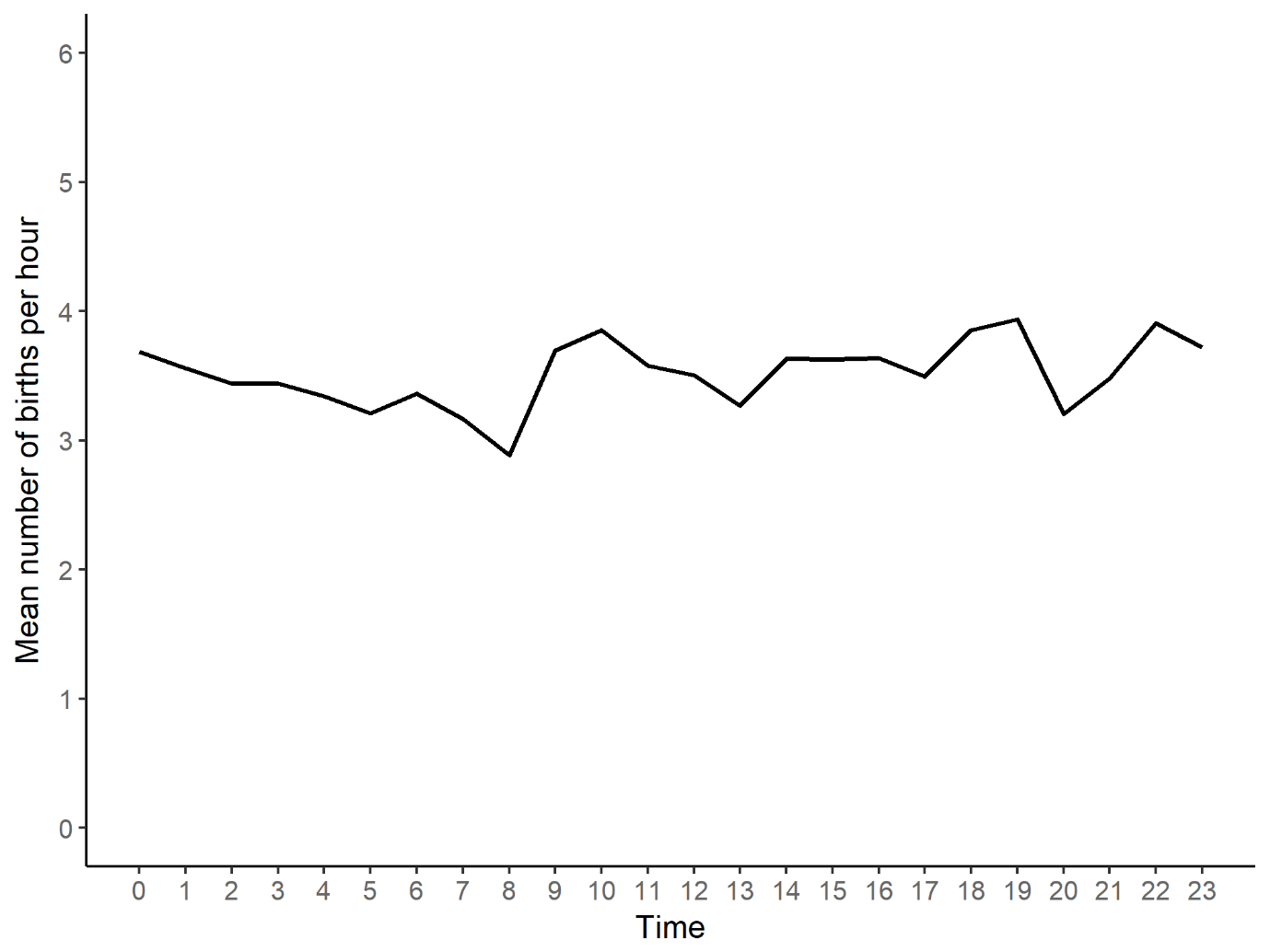
**

**Figure S4.4: Average number of births per hour on a non-holiday Thursday in NHS maternity units in England, 2005 – 2014: Induced labour, spontaneous birth**

**
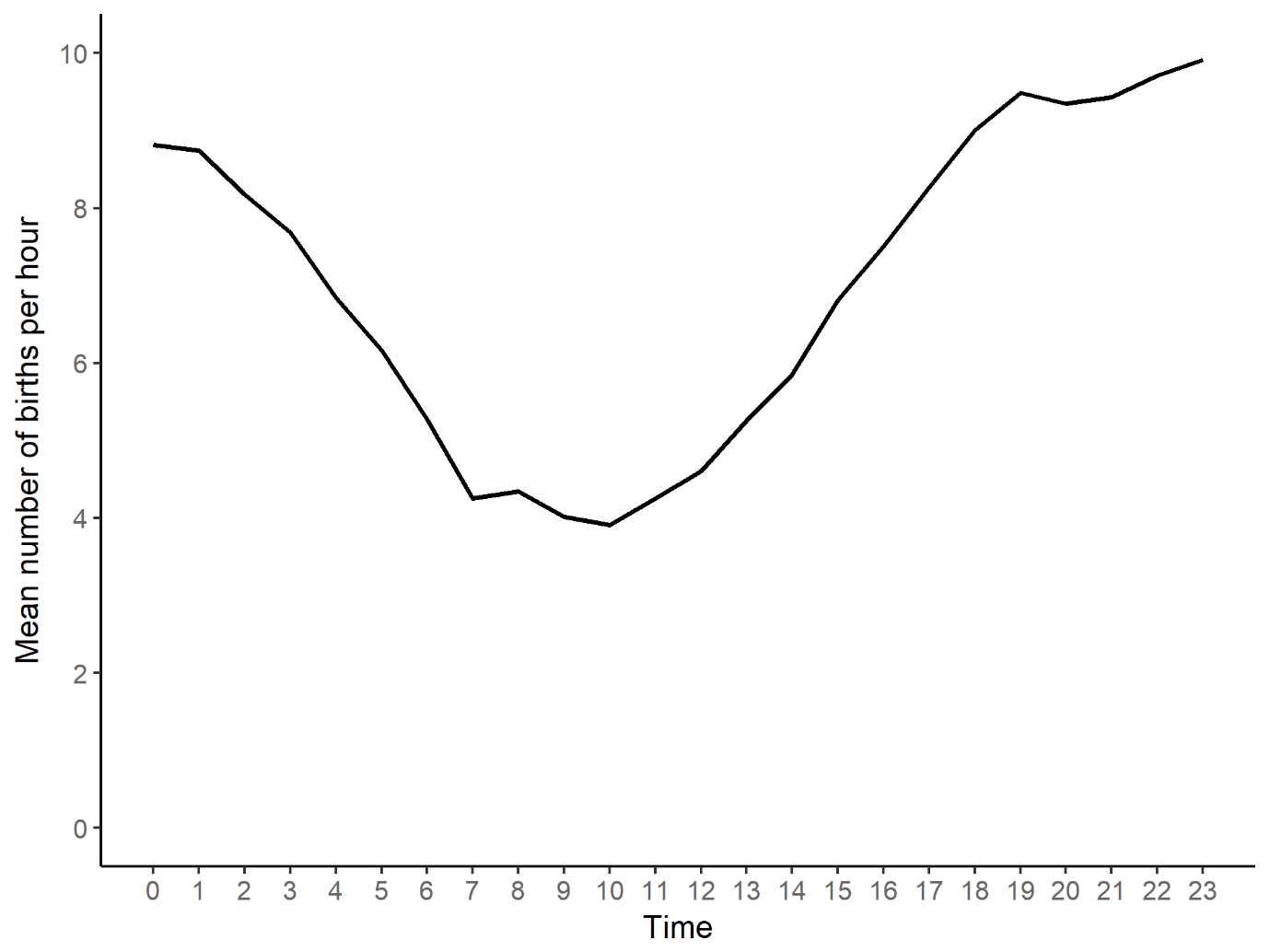
**

**Figure S4.5: Average number of births per hour on a non-holiday Thursday in NHS maternity units in England, 2005 – 2014: Induced labour, instrumental birth**

**
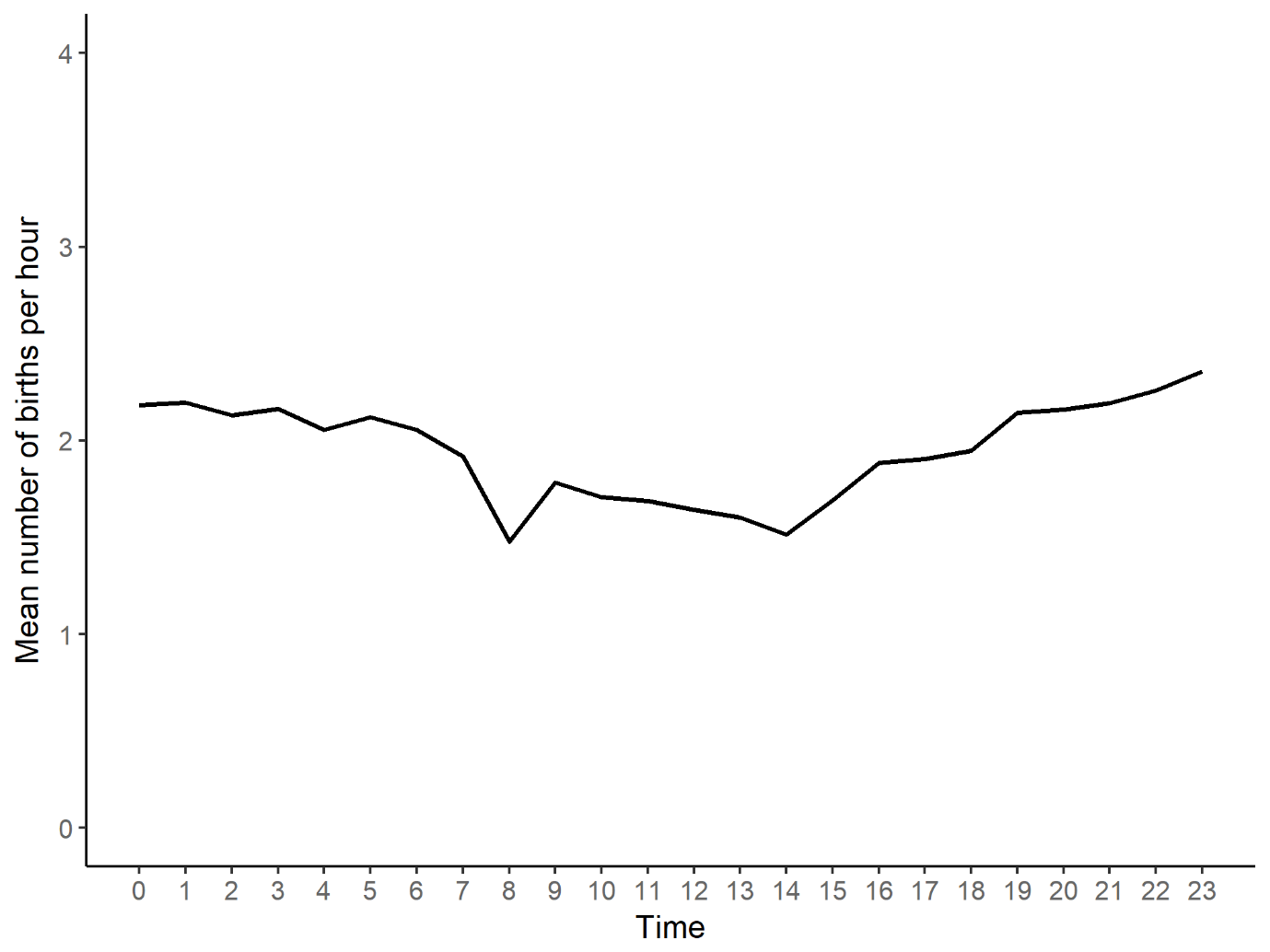
**

**Figure S4.6: Average number of births per hour on a non-holiday Thursday in NHS maternity units in England, 2005 – 2014: Induced birth, emergency caesarean**

**
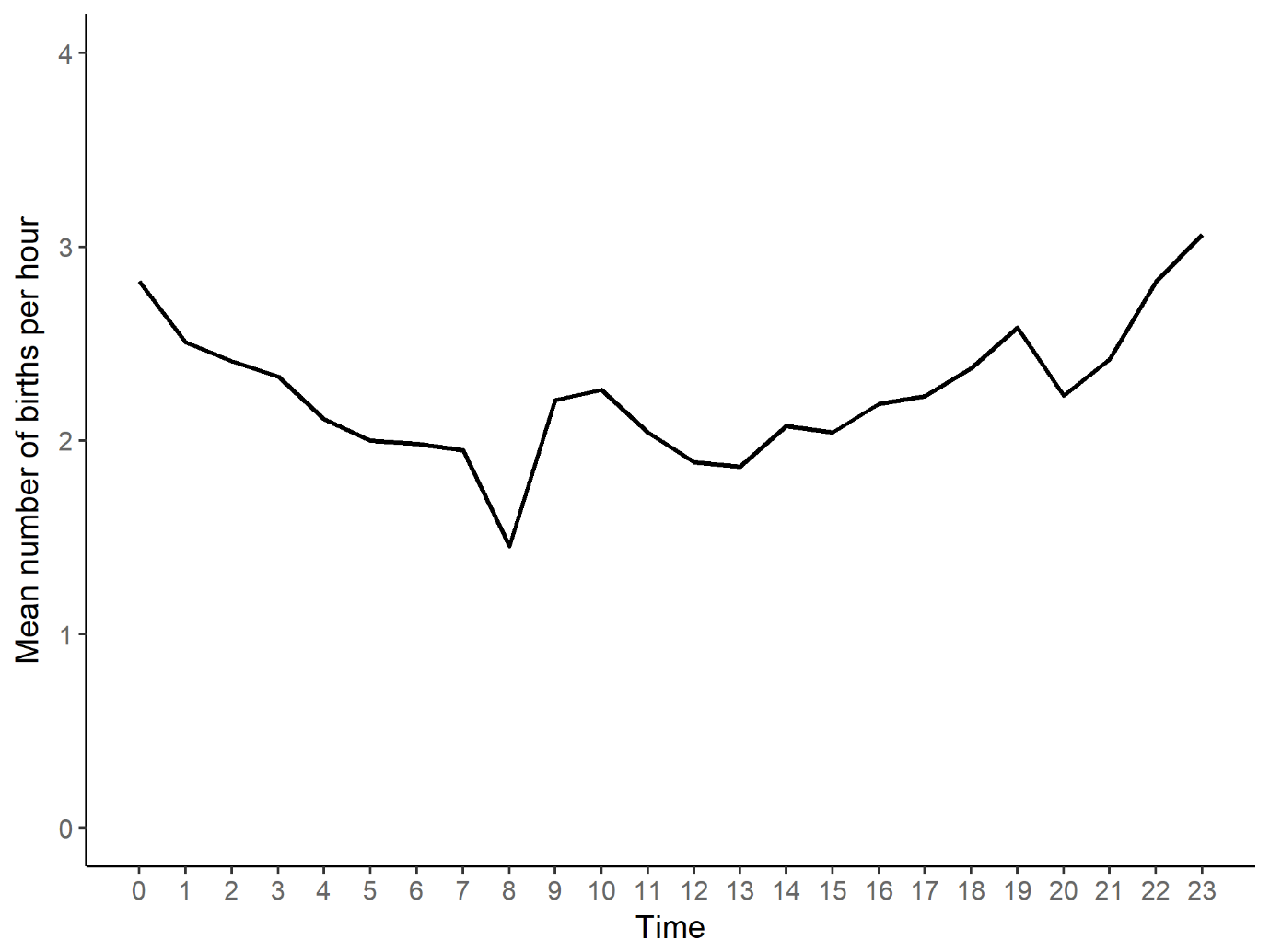
**

**Figure S4.7: Average number of births per hour on a non-holiday Thursday in NHS maternity units in England, 2005 – 2014: Elective caesarean**

**
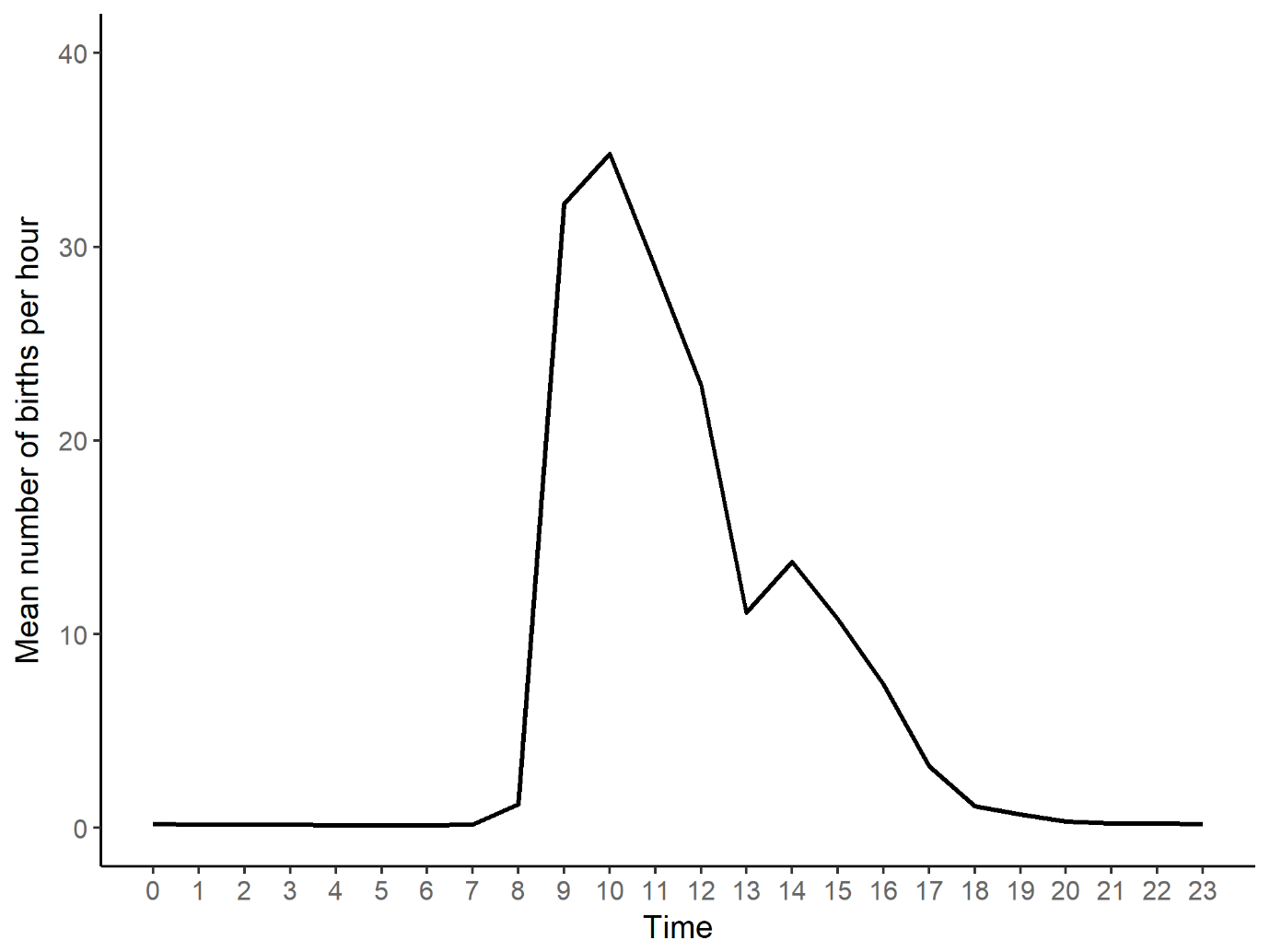
**

**Figure S4.8: Average number of births per hour on a non-holiday Thursday in NHS maternity units in England, 2005 – 2014: No labour, emergency caesarean**

**
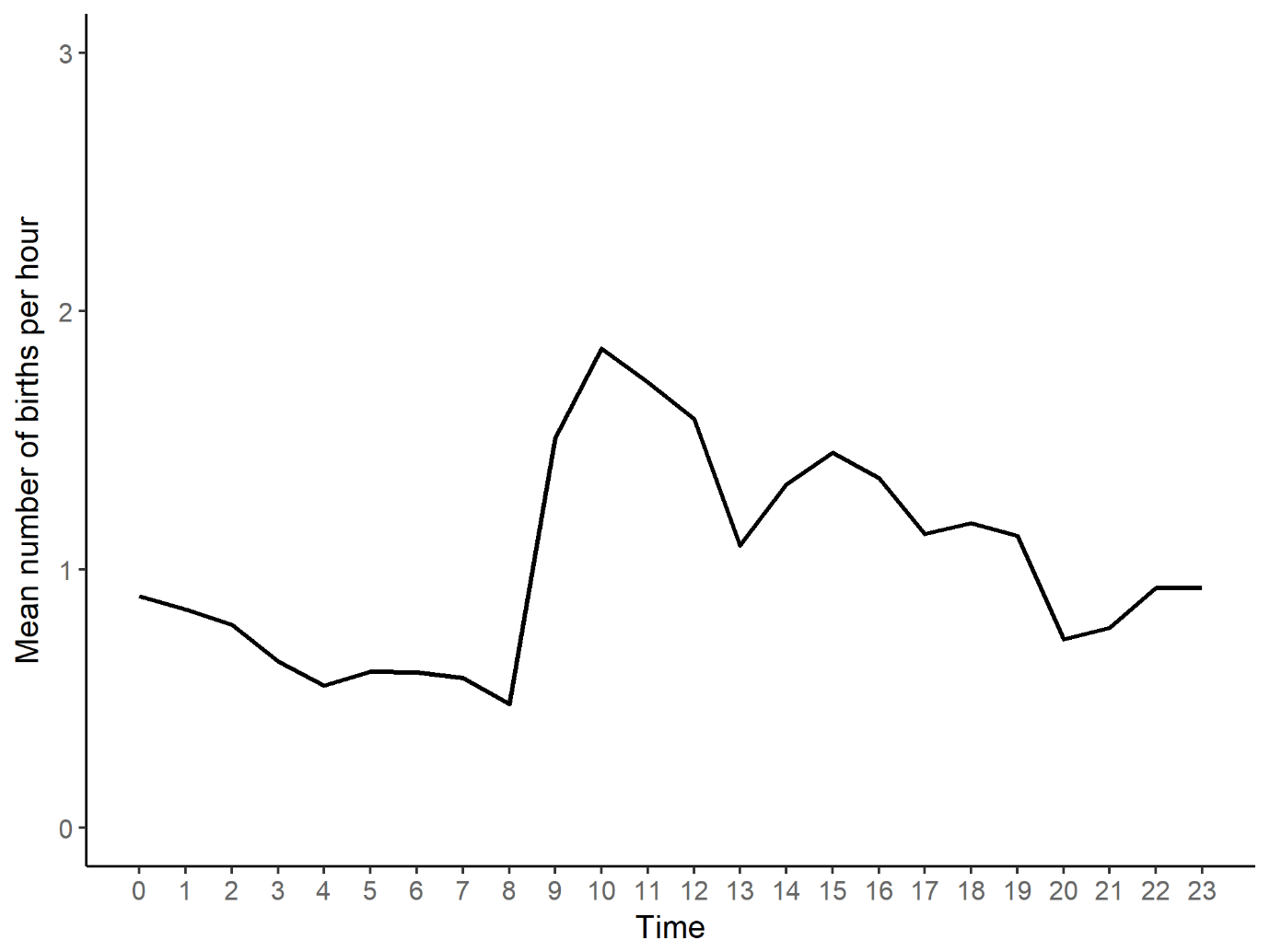
**
